# EAR domain-containing transcription factors trigger PRC2-mediated chromatin marking in Arabidopsis

**DOI:** 10.1093/plcell/koab139

**Published:** 2021-05-18

**Authors:** Fernando Baile, Wiam Merini, Inés Hidalgo, Myriam Calonje

**Affiliations:** Institute of Plant Biochemistry and Photosynthesis (IBVF-CSIC-US), Avenida Américo Vespucio 49, 41092, Seville, Spain

## Abstract

Polycomb group (PcG) complexes ensure that every cell in an organism expresses the genes needed at a particular stage, time, or condition. However, it is still not fully understood how PcG complexes PcG-repressive complex 1 (PRC1) and PRC2 are recruited to target genes in plants. Recent findings in *Arabidopsis thaliana* support the notion that PRC2 recruitment is mediated by different transcription factors (TFs). However, it is unclear how all these TFs interact with PRC2 and whether they also recruit PRC1 activity. Here, by using a system to bind selected TFs to a synthetic promoter lacking the complexity of PcG target promoters in vivo, we show that while binding of the TF VIVIPAROUS1/ABSCISIC ACID-INSENSITIVE3-LIKE1 recapitulates PRC1 and PRC2 marking, the binding of other TFs only renders PRC2 marking. Interestingly, all these TFs contain an Ethylene-responsive element binding factor-associated Amphiphilic Repression (EAR) domain that triggers both HISTONE DEACETYLASE COMPLEX and PRC2 activities, connecting two different repressive mechanisms. Furthermore, we show that different TFs can have an additive effect on PRC2 activity, which may be required to maintain long-term repression of gene expression.

## Teaser

The polycomb group complex PRC2 can be directed to target genes independently of PRC1 by EAR domain-containing transcription factors to mediate gene repression in Arabidopsis.

## Introduction

The evolutionarily conserved polycomb group (PcG) factors are required to maintain gene repression (Ringrose and Paro, 2004; Merini and Calonje, 2015). These factors form multiprotein complexes with different histone-modifying activities. PcG-repressive complex 1 (PRC1) has H2A E3 ubiquitin ligase activity toward lysine 119, 120, or 121 in fruit fly (*Drosophila melanogaster*), mammals, or *Arabidopsis thaliana*, respectively (Wang et al., 2004; Cao et al., 2005; Bratzel et al., 2010; Yang et al., 2013), and PRC2 has H3 lysine 27 (H3K27) trimethyltransferase activity (Müller et al., 2002; Makarevich et al., 2006; Mozgova and Hennig, 2015). Despite the conserved activity of these complexes, several studies indicate that distinct rules operate for PcG recruitment in different organisms (Müller and Kassis, 2006; Mendenhall et al., 2010; Xiao et al., 2017).

In Arabidopsis, PRC2 core subunits are well conserved compared to their animal counterparts (Mozgova et al., 2015); however, PRC1 composition is less conserved (Merini and Calonje, 2015). Although a H2A E3 ubiquitin ligase module containing one BMI1 (A, B, or C) and one RING1 (A or B) protein has been identified (Sanchez-Pulido et al., 2008; Bratzel et al., 2010), homologs for other PRC1 components are missing in Arabidopsis, and instead, several plant-specific proteins seem to function as PcG components (Calonje, 2014; Merini and Calonje, 2015). Distribution analysis of H2AK121ub and H3K27me3 peaks in Arabidopsis showed that both chromatin marks are generally targeted to gene regions, although H3K27me3 peaks are longer than H2AK121ub peaks. In addition, although H2AK121ub marks frequently co-localize with H3K27me3, there are also genes only marked with H3K27me3 or H2AK121ub (Zhou et al., 2017).

**Figure:**
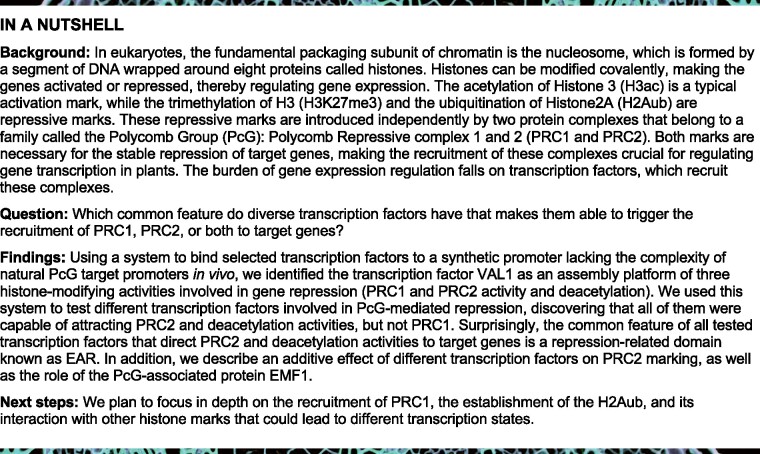


Regarding PcG recruitment in Arabidopsis, numerous transcription factors (TFs) have been related to PRC2 tethering. Among these are the *GAGA* motif binding proteins BASIC PENTACYSTEINE (BPC) 1–6 (Hecker et al., 2015; Xiao et al., 2017), the *TELOBOX* motif-binding proteins ARABIDOPSIS ZINC FINGER1 (AZF1), ZINC FINGER OF ARABIDOPSIS THALIANA6 (Xiao et al., 2017), and TELOMERE-REPEAT-BINDING FACTOR (TRB)1/2/3 (Zhou et al., 2018), the MYB TF ASYMMETRIC LEAVES1 (Lodha et al., 2013), the C2H2 TFs SUPERMAN (SUP; Xu et al., 2018) and KNUCKLES (KNU; Sun et al., 2019), and the MADS-box TFs FLOWERING LOCUS C (FLC) and SHORT VEGETATIVE PHASE (SVP; Wang et al., 2014; Richter et al., 2019). Furthermore, it was recently shown that certain genomic fragments located at several PcG targets, which contain binding sites for a wide variety of TF families, can recruit PRC2, thus functioning as Drosophila polycomb recruiting elements (Xiao et al., 2017). In addition, localization analyses of H2AK121ub and H3K27me3 marks in the wild-type (WT) and PcG Arabidopsis mutants showed that the levels of both H2AK121ub and H3K27me3 are substantially reduced in the PRC1 mutant *bmi1 abc* (Zhou et al., 2017), indicating that either the interaction with BMI1 or H2AK121ub mark is also important for PRC2 activity.

Unlike PRC2, the recruitment of the PRC1 H2A E3 ubiquitin ligase module has thus far only been associated with VIVIPAROUS1/*ABSCISIC ACID* INSENSITIVE3 (ABI3)-LIKE (VAL)1/2 proteins (Yang et al., 2013; Qüesta et al., 2016), which is surprising given the number of TFs involved in PRC2 recruitment and the apparent dependence of PRC1 for H3K27me3 marking. Thus, despite recent advances in understanding PcG recruitment in plants, there are still many unknowns. For instance, it is still unclear whether the recruitment of one complex requires the recruitment of the other, how PRC2 interacts with such a diverse group of TFs, and whether these interactions take place independently or in parallel. In addition, as there are genes marked with H2AK121ub/H3K27me3, H2AK121ub, or H3K27me3 (Zhou et al., 2017), it is unknown whether this differential marking depends on different factors and, in that case, if these factors can function simultaneously at some target genes.

Here, to address all these questions, we developed a system to mediate the binding of selected TFs to a synthetic promoter lacking the cis-regulatory elements involved in PcG recruitment in vivo, allowing us to investigate their roles under controlled conditions in Arabidopsis. Our results show that the binding of the TF VAL1 can recapitulate PRC1 and PRC2 marking and remove H3 acetylation (H3ac) marks from the synthetic locus. While PRC1 recruitment is mediated by a direct interaction with VAL1, PRC2 marking requires both PRC1 and VAL1. Interestingly, we found that PRC2 activity can be triggered independently of PRC1 by the binding of TFs from different families that contain an EAR domain as a common feature. We show that the EAR domain, possibly through its interaction with TOPLESS (TPL)/TPL-RELATED (TPR)1–4 corepressors or SIN3-ASSOCIATED POLYPEPTIDE18 (SAP18), connects HISTONE DEACETHYLASE (HDA) and PRC2 activities. Furthermore, we found that different TFs could have an additive effect on PRC2 activity, leading to increased levels of H3K27me3 at target genes. Our results not only unveil how the different PcG complexes can be directed to target genes, but also how different histone-modifying activities are coupled to promote gene repression in Arabidopsis.

## Results

### VAL1 acts as a platform for simultaneous assembly of PRC1, PRC2, and HDA activities

**Figure 1:**
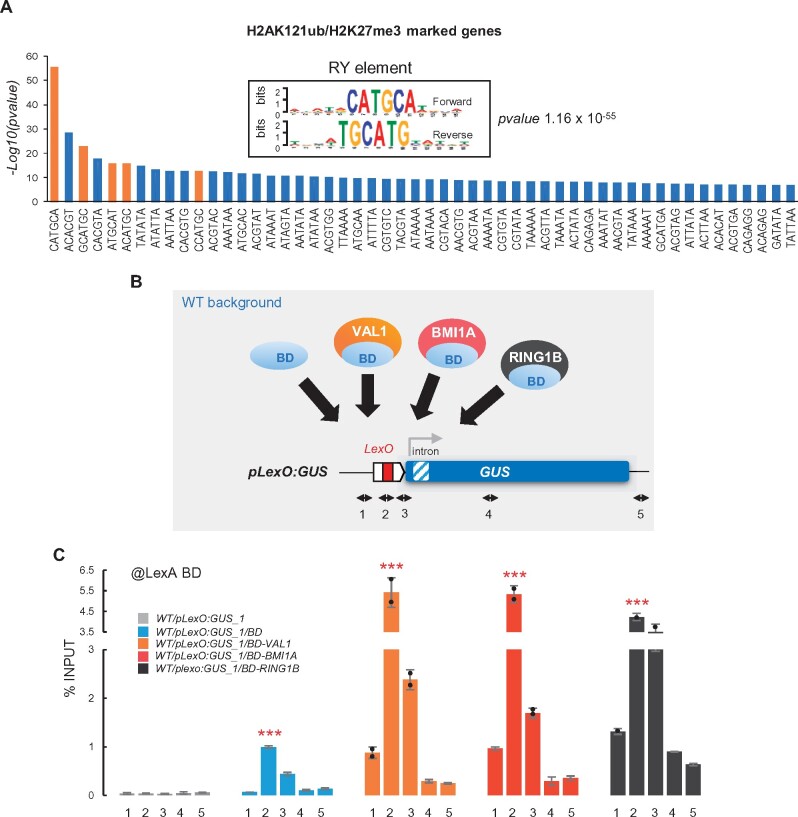
LexA BD fusion proteins bind to the synthetic promoter in vivo. A, Bar chart showing the RY element (indicated in orange) as the most significantly enriched cis-regulatory motif found at the 500 bp region upstream of the ATG of genes marked with H2AK121ub/H3K27me3 in WT that become upregulated in the *bmi1 abc* mutant (*n* = 1030 genes; see Supplemental Data Set S1). Analysis was carried out using TAIR Motif finder tool (https://www.arabidopsis.org/tools/bulk/motiffinder/index.jsp). Other significantly enriched 6-mer motifs are also shown. B, Schematic representation of the synthetic *GUS* reporter locus. The *LexO* element recognized by the LexA BD and the catalase intron are indicated. Numbered arrows indicate the positions of the primer pairs used to examine the binding of the fusion proteins to the synthetic locus. C, D, Bar charts showing BD, DB-VAL1, BD-BMI1A, and BD-RING1B enrichment at the *GUS* reporter locus determined by ChIP using anti-LexA BD antibody. WT/*pLexO:GUS_1* plants (-) were used as a negative control. Results are indicated as percentage of input. Error bars represent standard deviation of *n* = 2–3 independent pools of tissue. Significant differences at position 2 compared to control plants determined by two-sided Student’s *t* test are indicated (***P < 0.001)

The TFs VAL1/2 are involved in both PRC1- and PRC2-mediated repression (Yang et al., 2013; Qüesta et al., 2016; Chen et al., 2018; Jing et al., 2019; Zeng et al., 2020; Yuan et al., 2021). VAL TFs contain a B3 DNA-binding domain that specifically recognizes RY elements (CATGCA) (Suzuki et al., 2007). Therefore, we analyzed the 6-mer DNA motifs within the 500-bp regions upstream of the ATG in genes marked with H2AK121ub/H3K27me3 in the WT and upregulated in the PRC1 mutant *bmi1 abc* (Zhou et al., 2017; *n* = 1,030), finding that these elements were more highly enriched than other motifs (Figure 1A; Supplemental Data Set S1).

In addition, VAL1/2 interact with HDAs (Zhou et al., 2013; Zeng et al., 2020). Besides the B3 domain, VAL1/2 contains a plant homeodomain-like (PHD-L) domain, a Cysteine and tryptophan (CW) domain, and an EAR domain (Suzuki and McCarty, 2008). The PHD-L is involved in the homo- or heterodimerization of VAL1 and VAL2 (Chen et al., 2020), but it has also been proposed to act as a reader of H3 methylation states, like the CW domain (Hoppmann et al., 2011; Yuan et al., 2016). On the other hand, the EAR domain is involved in the interaction with TPL/TRP1-4 or SAP18, which in turn recruit HDA activities (Kagale and Rozwadowski, 2011). Nevertheless, although VAL1/2 can interact with these different repressive complexes, it is not clear whether these interactions take place simultaneously or within different contexts.

To investigate this issue, we developed a system to direct VAL1 recruitment to a constitutive promoter that lacks any of the cis-regulatory elements proposed to recruit PcG activity, including RY elements. For this, we built a synthetic target promoter consisting of a *cauliflower mosaic virus* (*CaMV35S*) promoter in which the bacterial LexA *operator* (*LexO*) was inserted. This promoter was placed upstream of the *beta-glucuronidase* (*GUS*) reporter gene, obtaining the *pLexO:GUS* construct (Figure 1B). In parallel, we generated a construct to constitutively express a translational fusion between the LexA DNA-binding domain (BD) and VAL1, and another to express the BD alone as a control (Figure 1B). In addition, since we wondered whether the synthetic tethering of the E3 ubiquitin ligase BMI1A or RING1B to DNA would lead to H2AK121 monoubiquitination and PRC2 activity, we generated the BD-BMI1A and BD-RING1B constructs using the same strategy (Figure 1B). All of these constructs were independently transformed into WT Col-0 Arabidopsis plants.

GUS activity was measured in different T1 *WT/pLexO:GUS* lines using a fluorometric assay (Supplemental Figure S1). All analyzed T1 lines were positive for GUS activity, although we found some variability in activity levels, most likely due to differences in the insertion sites of the transgene and/or the presence of more than one functional transgenic locus. We selected two different *WT/pLexO:GUS* lines (_1 and _2) with a 3:1 segregation ratio and verified *GUS* transcription in the corresponding homozygous seedlings (Supplemental Figure S1). In addition, we analyzed GUS activity levels in independent seedlings from the two different lines, finding similar levels among seedlings of the same line and between lines (Supplemental Figure S1). Moreover, GUS staining showed a similar localization pattern of GUS activity in the two lines (Supplemental Figure S1).

We also selected one T1 line of *WT/BD*, *WT/BD-VAL1*, *WT/BD-BMI1A*, or *WT/BD-RING1B* transgenic plants segregating for a single transgenic locus (3:1) and investigated protein expression in crude extracts of homozygous plants by immunoblot analysis using anti-LexA BD antibody (Supplemental Figure S2). Despite some differences in protein levels among the different lines, we confirmed that all proteins were overexpressed well above their native levels, and therefore, these differences should not pose a problem when comparing their effects on the synthetic locus. All these transgenic plants displayed a WT-like phenotype, indicating that overexpressing the fusion proteins did not interfere with the functions of the endogenous proteins. Next, to avoid possible indirect effects caused by insertion of the reporter locus in a different chromatin environment, we crossed each overexpressing line to the WT/*pLexO:GUS_1* line. To verify the effects of the fusion proteins in a different chromatin environment, we also crossed plants expressing some of these constructs with the *WT/pLexO:GUS_2* line.

We investigated the binding of the fusion proteins to the synthetic promoter by chromatin immunoprecipitation (ChIP) using anti-LexA BD antibody (Figure 1C). Quantitative polymerase chain reaction (qPCR) analysis was then performed using primer pairs located at a region upstream of the synthetic promoter (position 1), *LexO* (position 2), 5′-flanking region of the reporter gene (position 3), gene body of the reporter gene (position 4), and 3′-flanking region of the reporter gene (position 5; Figure 1B). We found that all these proteins were significantly enriched at the region corresponding to *LexO* (position 2); however, the immunoprecipitation efficiency was much higher in the case of BD-VAL1, BD-BMI1A, or BD-RING1B than that of the BD alone. Since protein amount was not a limiting factor (Supplemental Figure S2), this could have occurred because VAL1, BMI1A, and RING1B can form homodimers (Satijn and Otte, 1999; Chen et al., 2020), thus increasing the immunoprecipitation efficiency. In support of this idea, the same result was obtained when analyzing the binding of some of these proteins in the *WT/pLexO:GUS_2* background (Supplemental Figure S2).

**Figure 2:**
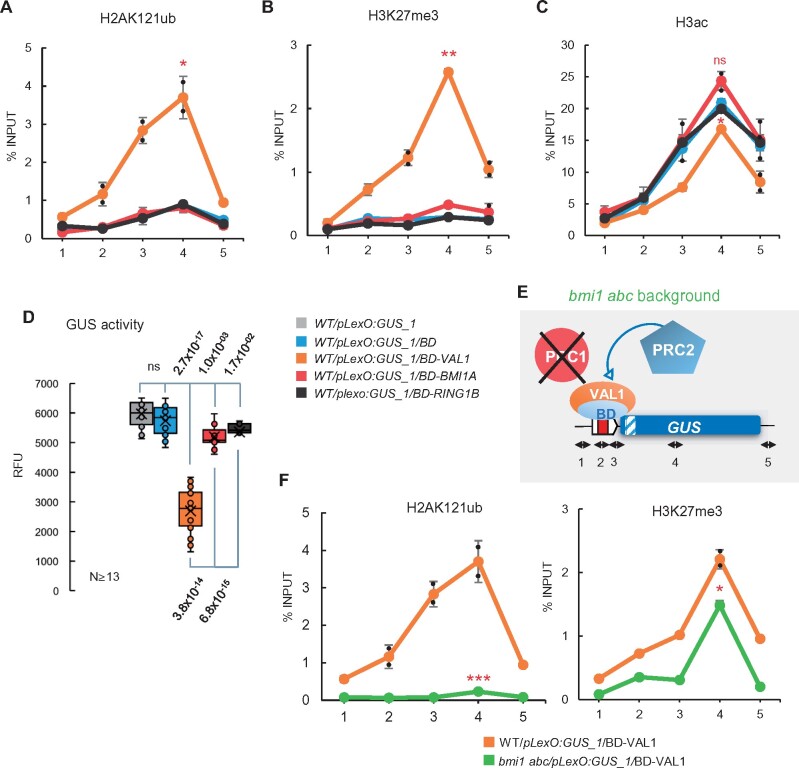
VAL1 directs PRC1, PRC2, and HDAC activities to target genes. A–C, H2AK121ub, H3K27me3, and H3ac levels at the *GUS* reporter locus in WT/*pLexO:GUS_1/BD*, WT/*pLexO:GUS_1/BD-VAL1*, WT/*pLexO:GUS_1/BD-BMI1A*, and WT/*pLexO:GUS_1/BD-RING1B* plants. Numbers at the *x*-axis indicate the positions of amplified regions as indicated in Figure 1B. Results are indicated as percentage of input. Error bars indicate standard deviation of *n* = 2 biological replicates. Significant differences at position 4 compared to WT/*pLexO:GUS_1/BD* are indicated as determined by two-sided Student’s *t* test (**P < 0.01; *P < 0.05; “ns” not significant). D, Box plot showing GUS activity levels in WT*/pLexO:GUS_1*, WT/*pLexO:GUS_1/BD*, WT/*pLexO:GUS_1/BD-VAL1*, WT/*pLexO:GUS_1/BD-BMI1A*, and WT/*pLexO:GUS_1/BD-RING1B* seedlings at 7 DAG. RFU indicates relative fluorescence units. Activity was tested in independent seedlings (*N* ≥ 13, combined from two independent experiments). In each case, the median (segment inside rectangle), the mean (cross inside the rectangle), upper and lower quartiles (boxes), and minimum and maximum values (whiskers) are indicated. *P*-value of differences between WT/*pLexO:GUS_1/BD* and the other plants determined by two-sided Student’s *t*-test are indicated. “ns” indicates not significant. E, F, Schematic representation of the experiment shown in F, in which the levels of H2AK121ub and H3K27me3 at the reporter locus were compared between WT/*pLexO:GUS_1/BD-VAL1* and *bmi1abc/pLexO:GUS_1/BD-VAL1* plants. Results are indicated as percentage of input. Error bars indicate standard deviation of *n* = 2 biological replicates. Significant difference at position 4 is indicated as determined by two-sided Student’s *t* test (***P < 0.001; *P < 0.05)

Next, we investigated whether H2AK121ub and H3K27me3 marks were incorporated at the synthetic locus in the different plants by ChIP using the corresponding antibodies. For this, we used the same primer pairs as for the localization of the fusion protein (Figure 1B). Since genome-wide distribution analysis of these marks showed that H2AK121ub and H3K27me3 are generally located at gene bodies (Zhou et al., 2017), the primer pair at position 4 should detect if these marks are present, although not necessarily where they display their highest enrichment. H2AK121ub and H3K27me3 marks were incorporated at the reporter gene in WT/*pLexO:GUS_1/BD-VAL1* but not in WT/*pLexO:GUS_1/BD*, WT/*pLexO:GUS_1/BD-BMI1A*, or WT/*pLexO:GUS_1/BD-RING1B* plants (Figure 2, A and B). This was also the case when we performed the experiments in the *WT/pLexO:GUS_2* background (Supplemental Figure S3).

To correct for possible differences in immunoprecipitation efficiency among samples, the ChIP results were normalized to the levels of these marks at *FLC*, which was used as an internal positive control for these modifications because its expression was not altered in transgenic plants (Supplemental Figure S4). The normalized data showed similar results (Supplemental Figure S5), indicating that while VAL1 was able to recapitulate PRC1 and PRC2 markings, the BD alone, BD-BMI1A, and DB-RING1B were not. The enrichment of BD-VAL1 at *LexO* and the detection of the histone modifications at the reporter gene body are consistent with the prevailing idea that PcG complexes are recruited to a specific regulatory region from which they incorporate their marks within gene bodies. The observation that we did not detect incorporation of H2AK121ub after the binding of BD-BMI1A or DB-RING1B indicates that these proteins were not functional under our experimental conditions. Either the fusion of BD to the N-terminal end of the proteins and/or their artificial binding to DNA hinders their E3 ubiquitin ligase activity, which is not surprising, as H2A monoubiquitination is a dynamic process that requires interaction with an E2 ligase and transference of the ubiquitin residue to a nucleosomal H2A substrate (Li et al., 2006). In any case, these negative results support the role of VAL1 in recruiting PRC1 and PRC2 activities and rule out the possibility that the differences observed were due to distinct binding efficiencies among proteins.

We also investigated the levels of H3ac marks at the reporter locus in the different lines, which are indicative of gene activation, by ChIP. The levels of H3ac marks were significantly reduced in WT/*pLexO:GUS_1/BD-VAL1* compared to WT/*pLexO:GUS_1/BD*, but not in WT/*pLexO:GUS_1/BD-BMI1A* or WT/*pLexO:GUS_1/BD-RING1B* plants (Figure 2C). Normalized ChIP data to the levels of H3ac at *ACTIN7* (*ACT7*), an internal positive control for this modification whose expression was not altered in transgenic plants (Supplemental Figure S4), showed similar results (Supplemental Figure S5). In addition, we examined the effects of these proteins on GUS activity. While the binding of lexA BD alone did not affect GUS activity levels compared to control *WT/pLexO:GUS_1* plants, the expression of BD-VAL1 led to a significant reduction of GUS activity (Figure 2D). Unexpectedly, even though the levels of H3ac at the reporter locus were not significantly affected in WT/*pLexO:GUS_1/BD-BMI1A* or WT/*pLexO:GUS_1/BD-RING1B* (Figure 2C), we observed a slight decrease in GUS activity after the binding of BD-BMI1A and BD-RING1B compared to control plants (Figure 2D). Similar results were obtained when analyzing *GUS* transcript levels (Supplemental Figure S6), suggesting that the binding of BD-BMI1A and BD-RING1B may interfere to some extent with transcription. In any case, these results indicate that VAL1 acts as a platform for the simultaneous assembly of different histone-modifying activities involved in gene repression.

Previous reports have shown that VAL1 and BMI1 proteins directly interact (Yang et al., 2013; Qüesta et al., 2016). Furthermore, we performed in vitro pull-down assays using BMI1A and different fragments of VAL1 protein, finding that the second half of VAL1 is required for this interaction (Supplemental Figure S7). In addition, since the levels of H3K27me3 are reduced in both *bmi1 abc* and *val1/2* mutants (Yang et al., 2013; Zhou et al., 2017; Yuan et al., 2021), we wondered whether VAL1 is directly involved in PRC2 recruitment or if this is mediated by PRC1. To investigate this notion, we introduced the *pLexO:GUS_1* and *BD-VAL1* transgenes into the *bmi1 abc* mutant background (Figure 2E) and analyzed the levels of H2AK121ub and H3K27me3 at the reporter locus (Figure 2F). In accordance with BMI1 activity, H2AK121ub marks were undetectable in *bmi1 abc*. In addition, we found reduced levels of H3K27me3 in *bmi1 abc* compared to WT. To confirm this result, H3K27me3 levels at the reporter locus were normalized to the levels of this modification at *AGAMOUS* (*AG*), which was used as internal control as the levels of H3K27me3 at this gene are not altered in *bmi1 abc* (Supplemental Figure S4). Normalized data also showed reduced levels of H3K27me3 in the *bmi1 abc* mutant (Supplemental Figure S5). Nevertheless, although H3K27me3 levels were significantly reduced in *bmi1 abc*, some of this mark was still detected, indicating that PRC2-mediated H3K27me3 marking may require interactions with both PRC1 and VAL1.

### The binding of other TFs triggers PRC2 and HDA activities but not H2AK121ub marking

**Figure 3:**
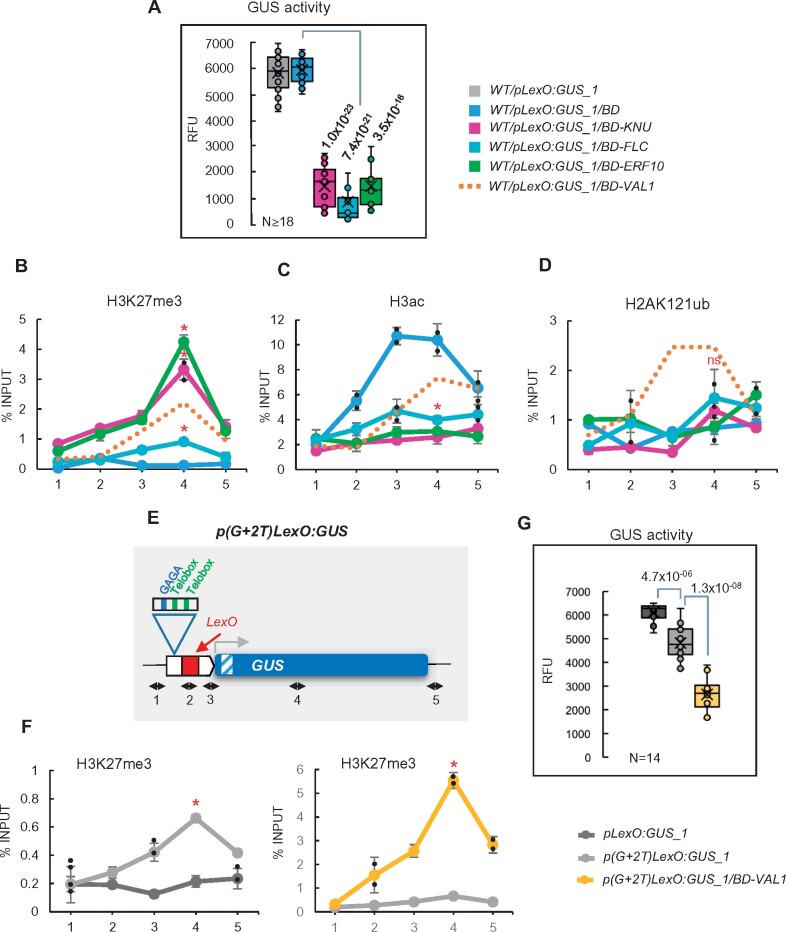
Synthetic tethering of BD-KNU, BD-FLC, or BD-ERF10 triggers PRC2 and HDAC activities but not PRC1 activity. A, Box plots showing GUS activity levels in WT/*pLexO:GUS_1*, WT/*pLexO:GUS_1/BD*, WT/*pLexO:GUS_1/BD-KNU*, WT/*pLexO:GUS_1/BD-FLC*, and WT/*pLexO:GUS_1/BD-ERF10* seedlings at 7 DAG indicated as RFU. Activity was tested in independent seedlings (*N* ≥ 18, combined from two independent experiments). In each case, the median (segment inside rectangle), the mean (cross inside the rectangle), upper and lower quartiles (boxes), and minimum and maximum values (whiskers) are indicated. *P*-value of differences between WT/*pLexO:GUS_1/BD* and the other plants determined by two-sided Student’s *t*-test are indicated. B–D, H3K27me3, H3ac, and H2AK121ub levels at the *GUS* reporter locus in different plants. Results are indicated as percentage of input. Numbers at the *x*-axis indicate the positions of amplified regions as indicated in Figure 1B. Error bars indicate standard deviation of *n* = 2 independent pools of tissue. Significant differences compared to WT/*pLexO:GUS_1/BD* determined by two-sided Student’s *t* test are indicated (*P < 0.05; “ns” not significant). One replicate of WT/*pLexO:GUS_1/BD-VAL1* was included as an additional control (orange dotted line). E, Schematic representation of the *p(G + 2T)LexO:GUS* construct in which one *GAGA* and two *TELOBOX* motifs were inserted upstream of the *LexO*. F, H3K27me3 levels at the *GUS* reporter locus in WT*/pLexO:GUS_1* and WT*/p(G + 2T)LexO:GUS_1* plants (left), and WT*/pLexO:GUS_1* and WT*/p(G + 2T)LexO:GUS_1*/BD-VAL1 (right). Results are indicated as percentage of input*.* Error bars indicate standard deviation of *n* = 2 independent pools of tissue. Significant differences at position 4 determined by two-sided Student’s *t* test are indicated (*P < 0.05). G, Box plot showing GUS activity levels in WT*/pLexO:GUS_1*, WT*/p(G + 2T)LexO:GUS_1* and WT*/p(G + 2T)LexO:GUS_1/BD-VAL1* plants. Activity was tested in *N* = 14 independent seedlings at 7 DAG. *P*-value of differences determined by two-sided Student’s *t* test are indicated

A broad diversity of TFs belonging to different gene families is able to bind to PRE-like sequences in Arabidopsis (Xiao et al., 2017). Among the most highly enriched are the C2H2 and AP2-ERF family TFs (Xiao et al., 2017). Accordingly, several studies suggest that the C2H2 TFs SUP, KNU, and AZF1 are able to recruit PRC2 activity (Xiao et al., 2017; Xu et al., 2018; Sun et al., 2019). The MADS-box TFs FLC and SVP have also been connected to PRC2 repression (Wang et al., 2014; Richter et al., 2019). Therefore, we wondered whether all these TFs, like VAL1, were able to function as assembly platforms for PRC1, PRC2, and possibly other histone-modifying complexes. To test this, we generated BD-KNU, BD-FLC, and BD-ERF10 fusions and analyzed their effects on our synthetic target locus. Even though the three proteins were overexpressed (Supplemental Figure S8), we did not find alterations in the phenotypes of the transgenic plants compared to WT, which was surprising, as one would expect that overexpressing these proteins would cause developmental changes. However, we found that GUS activity was significantly reduced in the three transgenic lines (Figure 3A), indicating that these fusion proteins were functional when recruited to the reporter locus. Furthermore, we found that the binding of the three proteins led to the incorporation of significant levels of H3K27me3 and the removal of H3ac marks (Figure 3, B and C show results as percentage of input and Supplemental Figure S9 normalized data to an internal control; see Supplemental Figure S8 for the expression of control genes in transgenic plants). However, we did not detect incorporation of H2AK121ub marks (Figure 3D; Supplemental Figure S9). Consistent with these results, we found that the cis-regulatory motifs recognized by these TFs were enriched in the 500-bp region upstream of the ATG of the genes marked only with H3K27me3 (Zhou et al., 2017; Supplemental Figure S10 and Supplemental Data Set S1). All together, these results support the notion that PRC2 and HDA activities can be triggered by different TFs, while the incorporation of H2AK121ub marks requires VAL TFs.

Nevertheless, as the 500-bp region upstream of the ATG of H2AK121ub/H3K27me3-marked genes showed enrichment in other cis-regulatory motifs in addition to RY elements (Figure 1A; Supplemental Data Set S1), we wondered whether different TFs could function simultaneously in PRC2 marking. Since introducing two different BD fusion proteins in the same plant could result in competition for the binding site, we reasoned that inserting a DNA fragment containing the binding motifs of TFs previously associated with PcG recruitment into the synthetic promoter could help us address this question. We selected the *GAGA* and *TELOBOX* motifs, as the binding of BPC1, AZF1, and TRB1/2/3 to these motifs has been shown to promote the recruitment of PRC2 (Hecker et al., 2015; Xiao et al., 2017; Zhou et al., 2018). Although this experiment relied on the binding of endogenous and overexpressed TFs, whose expression levels may differ and whose expression patterns may not always overlap, we wanted, in any case, to investigate if this had any effect on the incorporation of H3K27me3 at the reporter locus. Therefore, we introduced a fragment of DNA containing one *GAGA* and two *TELOBOX* motifs upstream of the *LexO* into the reporter construct to generate WT/*p(G + 2T)LexO:GUS* lines (Figure 3E; Supplemental Figure S11).

We first compared GUS activity levels in different *WT/pLexO:GUS* and WT*/p(G + 2T)LexO:GUS* T1 lines, finding that GUS activity was significantly reduced in WT*/p(G + 2T)LexO:GUS* compared to the *WT/pLexO:GUS* lines (Supplemental Figure S12). We then selected one WT*/p(G + 2T)LexO:GUS* line segregating for a single insertion (WT*/p(G + 2T)LexO:GUS_1*). We considered the possibility that the decrease in GUS activity in WT*/p(G + 2T)LexO:GUS* compared to *WT/pLexO:GUS* could be due to impaired performance of the promoter after the insertion of an extra DNA fragment. However, when comparing the levels of H3K27me3 in WT*/p(G + 2T)LexO:GUS_1* and WT*/pLexO:GUS_1*, we detected significantly increased levels of these marks in WT*/p(G + 2T)LexO:GUS_1* (Figure 3F left panel; Supplemental Figure S9 shows normalized data to an internal control), supporting the notion that TFs recognizing these motifs could promote PRC2 recruitment.

We then checked the levels of H3K27me3 at the *p(G + 2T)LexO:GUS_1* reporter locus after the binding of BD-VAL1, finding significantly increased levels of these marks in WT/*p(G + 2T)LexO:GUS_1/BD-VAL1* compared to WT/*p(G + 2T)LexO:GUS_1* (Figure 3F right panel; Supplemental Figure S9 shows normalized data to an internal control). Consistent with this finding, GUS activity was significantly lower in *p(G + 2T)LexO:GUS_1/BD-VAL1* than in WT/*p(G + 2T)LexO:GUS_1* plants (Figure 3G), indicating that different TFs can have an additive effect on PRC2 marking.

### The EAR domain contributes to direct PRC2 activity to target genes

**Figure 4:**
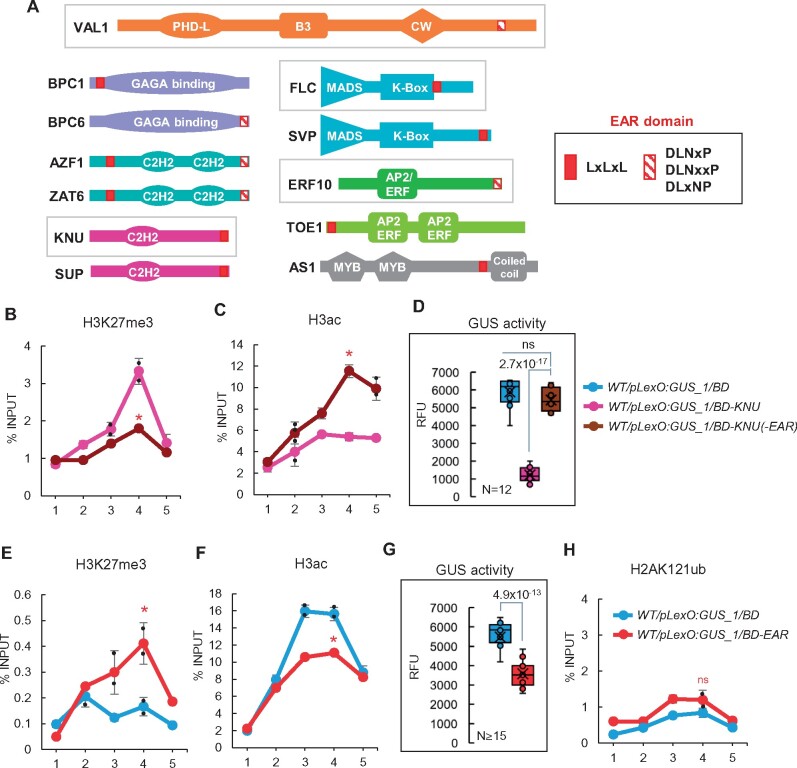
The EAR domain connects PRC2 marking and H3 deacetylation. A, Schematic representation of the domains present at TFs related to PRC2 recruitment. The TFs analyzed in this work are indicated. B, C, Comparison of H3K27me3 and H3ac levels at the *GUS* reporter locus in WT/*pLexO:GUS_1/BD-KNU* and WT/*pLexO:GUS_1/BD-KNU(-EAR)* plants. Numbers at the *x*-axis indicate the positions of amplified regions as indicated in Figure 1B. Results are indicated as percentage of input. Error bars indicate standard deviation of *n* = 2 independent pools of tissue. Significant differences at position 4 determined by two-sided Student’s *t* test are indicated (*P < 0.05). D, Box plot showing GUS activity levels in WT/*pLexO:GUS_1/BD*, WT/*pLexO:GUS_1/BD-KNU*, and WT/*pLexO:GUS_1/BD-KNU(-EAR)* seedlings at 7 DAG indicated as RFU. Activity was tested in independent seedlings (*N* ≥ 12, combined from two independent experiments). The median (segment inside rectangle), the mean (cross inside the rectangle), upper and lower quartiles (boxes), and minimum and maximum values (whiskers) are indicated. *P*-value of differences determined by two-sided Student’s *t* test are indicated. “ns” indicates not significant. E, F, H3K27me3 and H3ac levels at the *GUS* reporter locus in WT/*pLexO:GUS_1/BD* and WT/*pLexO:GUS_1/BD-EAR* plants. Results are indicated as percentage of input. Error bars indicate standard deviation of *n* = 2 independent pools of tissue. Significant differences between WT/*pLexO:GUS_1/BD* and WT/*pLexO:GUS_1*/*BD-EAR* determined by two-sided Student’s *t* test are indicated (*P < 0.05). G, Box plot showing GUS activity levels in the same plants indicated as RFU. Activity was tested in independent seedlings (*N* ≥ 15, combined from two independent experiments). *P*-value of difference between WT/*pLexO:GUS_1/BD* and WT/*pLexO:GUS_1/BD-EAR* plants determined by two-sided Student’s *t* test are indicated. H, H2AK121ub levels at the reporter locus in the different plants. Results are indicated as percentage of input. Error bars indicate standard deviation of *n* = 2 independent pools of tissue. No significant differences (“ns”) were detected according to two-sided Student’s *t* test

Since all the TFs tested, including VAL1, were able to direct PRC2 activity to the synthetic promoter, we examined if they display some common feature. Interestingly, despite the lack of sequence homology among them, these TFs contain an EAR domain. Furthermore, except for TRB TFs, all TFs related to PRC2 recruitment before have been shown to contain an EAR domain (Suzuki and McCarty, 2008; Xiao et al., 2017; Xu et al., 2018; Sun et al., 2019; Richter et al., 2019; Figure 4A). The EAR domain is defined as LxLxL, DLNxP, and DLNxxP. This domain has been found in many TFs of different gene families with repressive activity, constituting what has been named the EAR repressome (Kagale et al., 2010). The EAR domain mediates interactions with TPL/TPR corepressors or SAP18 protein (Song and Galbraith, 2006; Kagale and Rozwadowski, 2011; Causier et al., 2012). TPL/TPR can interact with the HDAs HDA6 and HDA19 (Liu et al., 2014), and importantly, with the PcG proteins EMBRYONIC FLOWER1 (EMF1) and VERNALIZATION5 (VRN5; Causier et al., 2012; Ke et al., 2015; Collins et al., 2019). SAP18 is both a component of SIN3-HISTONE DEACETYLASE COMPLEXES (HDACs; Zhang et al., 1997) and the APOPTOSIS AND SPLICING-ASSOCIATED PROTEIN complex (Deka and Singh, 2017). The SIN3-HDACs in Arabidopsis includes a SIN3-like protein (SNL1–6), SAP18, SAP30, one HDA protein (HDA19, HDA9, HDA7, or HDA6), and MULTICOPY SUPPRESSOR OF IRA1 (MSI1; Liu et al., 2014), which is also a PRC2 core component (Derkacheva et al., 2013; Mehdi et al., 2016; Ning et al., 2019). Moreover, SAP18 co-purifies with different PRC2 core components and HDA19 (Qüesta et al., 2016). All together, these data strongly suggest that there is a direct connection between EAR-containing TFs yriTPL/TPR-HDACs or SAP18-HDACs and PRC2, a concept that requires further investigation.

Therefore, to explore whether the EAR domain could trigger PRC2-mediated H3K27 trimethylation in addition to H3 deacetylation, we generated a mutated BD-KNU version in which the EAR domain was removed (BD-KNU(-EAR); Supplemental Figure S13) and compared the levels of H3K27me3 marks and H3ac at the reporter locus after the binding of BD-KNU or BD-KNU(-EAR). The levels of H3K27me3 were significantly reduced in *pLexO:GUS_1/BD-KNU(-EAR)* compared to WT/*pLexO:GUS_1/BD-KNU*, whereas the opposite effect was observed for H3ac marks (Figure 4, B and C; Supplemental Figure S14 shows normalized data to an internal control; see Supplemental Figure S13 for the expression levels of control genes in these plants)*.* Furthermore, GUS activity levels in *pLexO:GUS_1/BD-KNU(-EAR)* were similar to those in WT/*pLexO:GUS_1/BD* plants (Figure 4D).

To further verify these results, we checked if the BD fused to an EAR domain (BD-EAR) (Supplemental Figure S15) could increase H3K27me3 levels and reduce H3ac levels at the reporter locus. Indeed, we found that the EAR domain was able to cause these effects (Figure 4, E and F; Supplemental Figure S15 shows normalized data to an internal control). In addition, GUS activity was reduced in WT/*pLexO:GUS_1/BD*-*EAR* compared to WT/*pLexO:GUS_1/BD* (Figure 4G). Conversely, BD-EAR was unable to attract PRC1 for H2AK121 monoubiquitination (Figure 4H; Supplemental Figure S15 shows normalized data to an internal control), supporting the notion that the EAR domain is involved in directing both HDAC and PRC2 activities to target genes.

### EMF1–TPL interaction couples H3K27me3 marking to H3 deacetylation

**Figure 5:**
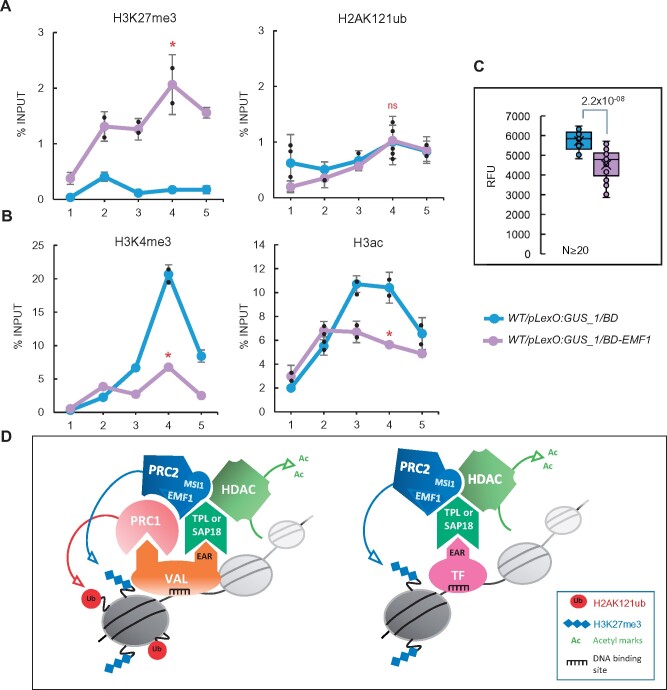
EMF1 recruitment results in the incorporation of H3K27me3 and the removal of H3K4me3 and H3ac. A, H3K27me3 and H2AK121ub levels at the *GUS* reporter locus in WT/*pLexO:GUS_1/BD* and WT/*pLexO:GUS_1/BD-EMF1* plants. Numbers at the *x*-axis indicate the positions of amplified regions as indicated in Figure 1B. Results are indicated as percentage of input. Error bars indicate standard deviation of *n* = 2–3 biological replicates. Significant differences at position 4 compared to WT/*pLexO:GUS_1/BD* are indicated as determined by two-sided Student’s *t* test (*P < 0.05; “ns” indicates not significant). B, H3K4me3 and H3ac levels at the *GUS* reporter locus in the different plants. Results are indicated as percentage of input. Error bars indicate standard deviation of *n* = 2 independent pools of tissue. Significant differences at position 4 are indicated as determined by two-sided Student’s *t* test (*P < 0.05). C, Box plots showing GUS activity levels in WT/*pLexO:GUS_1/BD* and WT/*pLexO:GUS_1/BD-EMF1* seedlings at 7 DAG. RFU indicates relative fluorescence units. Activity was tested in independent seedlings (*N* ≥ 20, combined from two independent experiments). In each case, the median (segment inside rectangle), the mean (cross inside the rectangle), upper and lower quartiles (boxes), and minimum and maximum values (whiskers) are indicated. *P*-value of differences between WT/*pLexO:GUS_1/BD* and WT/*pLexO:GUS_1/BD-EMF1* plants determined by two-sided Student’s *t* test are indicated. D, Drawing summarizing the histone modifying complexes recruited by VAL1 or by other EAR-containing TFs to promote transcriptional repression

We also used our system to investigate the role of the plant-specific PcG-associated factor EMF1 (Calonje et al., 2008). EMF1 was proposed to be a PRC1 component due to its ability to perform similar functions to those of Drosophila Psc in vitro (Calonje et al., 2008; Bratzel et al., 2010; Beh et al., 2012). However, several studies indicate that EMF1 is required for H3K27me3 marking at some PcG target genes (Calonje et al., 2008; Kim et al., 2012; Li et al., 2018). Accordingly, EMF1 interacts with MSI1 (Calonje et al., 2008) and co-purifies with PRC2 components (Liang et al., 2015). In addition, EMF1 interacts with FLC and the histone demethylase JMJ14, which removes H3K4me3 marks to mediate repression of the florigen gene *FT* (Wang et al., 2014). On the contrary, EMF1 seems to be dispensable for PRC1 marking, as the levels of this modification are not altered genome-wide in *emf1* (Yin et al., 2021). Thus, we analyzed the levels of H3K27me3 and H2AK121ub at the reporter locus after the binding of BD-EMF1 to the synthetic promoter. In agreement with previous results, we found that the levels of H3K27me3 were higher in WT/*pLexO:GUS_1/BD-EMF1* compared to WT/*pLexO:GUS_1/BD* plants, whereas those of H2AK121ub were not significantly altered (Figure 5A; Supplemental Figure S16 shows normalized data to an internal control; see Supplemental Figure S17 for the expression levels of control genes in these plants). These results confirm the notion that EMF1 is required for H3K27me3 marking but not for H2AK121 monoubiquitination.

Supporting the direct interaction of EMF1 and JMJ14 (Wang et al., 2014), the levels of H3K4me3 marks were reduced at the reporter locus in WT/*pLexO:GUS_1/BD-EMF1* compared to WT/*pLexO:GUS_1/BD* (Figure 5B; Supplemental Figure S16 shows normalized data to an internal control). Besides, EMF1 was shown to interact with TPL in a yeast two hybrid assay (Causier et al., 2012). Moreover, TPL was found among the proteins that co-immunoprecipitated with EMF1 (Bloomer et al., 2020; Supplemental Data Set S2). Consistent with this finding, we detected reduced levels of H3ac at the reporter locus after EMF1 binding (Figure 5B; Supplemental Figure S16 shows normalized data to an internal control). We then analyzed the levels of GUS activity in WT/*pLexO:GUS_1/BD-EMF1*, finding decreased levels compared to control plants (Figure 5C). All together, these data support a PRC2–EMF1–TPL–HDAC interaction.

## Discussion

PcG complexes ensure that each cell in an organism expresses the genes that are needed at a particular stage, time, or condition. It has been recently shown that PcG proteins mediate gene repression by regulating chromatin accessibility (Yin et al., 2021). However, as PcG proteins do not have the ability to recognize DNA sequences, how PRC1 and PRC2 activities are recruited to the appropriate target gene is still not fully understood. Recent data support the notion that PRC2 is recruited to its targets via interactions with different TFs. However, whether these TFs display a common feature, whether the same TF can recruit PRC2 and PRC1, and how PcG-mediated differential marking is achieved are currently unknown. In this study, we were able to dissect how PRC1 and PRC2 activities are directed to target genes in Arabidopsis.

We found that the binding of the TF VAL1 was able to recapitulate PRC1 and PRC2 marking and to couple HDA activities, acting as an assembly platform for different repressive complexes (Figure 5D, left panel). While PRC1 recruitment is mediated by the direct interaction of BMI1 with VAL1 (Yang et al., 2013; Qüesta et al., 2016), PRC2 activity requires both PRC1 and VAL1. Interestingly, our data show that although other TFs such as KNU, FLC, or ERF10 can trigger H3K27 trimethylation and H3 deacetylation to target genes, they cannot attract PRC1 for H2AK121ub marking (Figure 5D, right), indicating that PRC2 activity is directed to target genes through a more general mechanism than PRC1.

When comparing the protein domains present in VAL1, KNU, FLC, ERF10, and other TFs that have been related to PRC2 recruitment before, we found that, except for TRB1/2/3 factors, the only common feature among them is that they contain an EAR domain. Interestingly, unlike the other TFs, TRB1/2/3 factors seem to be stable PRC2-accesory proteins, as they co-purify with PRC2 (Bloomer et al., 2020).

The EAR domain interacts with TPL/TPR or SAP18, which in turn recruit HDA activities (Song and Galbraith, 2006; Kagale et al., 2010; Kagale and Rozwadowski, 2011; Causier et al., 2012). Interestingly, TPL and SAP18 also interact with PcG proteins. In fact, TPL co-purifies with EMF1 (Bloomer et al., 2020) and SAP18 with MSI1 (Mehdi et al., 2016), suggesting that they participate in HDAC and PRC2 assembly. In support of this notion, we found that the binding of three EAR-containing TFs led to the incorporation of H3K27me3 and the removal of H3ac marks at the reporter locus, and that this requires the EAR domain, as BD–EAR fusion protein by itself was able to cause these effects. Nevertheless, we observed that the efficiency of the BD–EAR fusion protein was lower than that of BD-KNU, BD-FLC, and BD-ERF10 TFs. A direct interaction of different PRC2 components with some of these EAR-containing TFs has been also reported. For instance, yeast-two-hybrid assays using EMF2, CLF, or FIE as bait and AZF1, BPC1, or TOE1 full-length proteins or protein fragments as prey revealed interactions between these proteins (Xiao et al., 2017). Nonetheless, no single protein motif appears to be responsible for these interactions (Xiao et al., 2017), suggesting that different contacts between TFs and PcG proteins may reinforce PRC2 recruitment. Consistent with this notion, the in vivo interaction of KNU with FIE involves both the C2H2 zinc finger and EAR domains (Sun et al., 2019). Similarly, the interaction of SUP with CLF but not with LHP1 requires the intact EAR domain (Xu et al., 2018). Moreover, our results show that the recruitment of EMF1 to the synthetic promoter leads to H3K27me3 incorporation and H3ac removal. Since EMF1 directly interacts with TPL/TPR and MSI1 with SAP18, we propose that the EAR-containing TFs act as an anchoring point for the recruitment of PRC2 and HDACs via interactions with TPL/TPR or SAP18 (Figure 5).

On the other hand, it is unknown whether the interactions of the EAR-containing TFs with TPL/TRP or SAP18 depend on the type of EAR domain or the presence of adjacent DNA sequences. It is also unknown whether TPL/TRP and SAP18 functionally overlap, as some of EAR-containing TFs have been reported to interact with both proteins (Kagale et al., 2010; Kagale and Rozwadowski, 2011; Causier et al., 2012). In any case, since TPL/TRP and SAP18 are expressed in most plant tissues (Kagale and Rozwadowski, 2011), the ability of the PcG machinery to maintain specific transcriptional states in different cell types, at different times, or under different conditions may rely on the EAR-containing recruiting factors, whose expression is tightly regulated in response to internal and external signals.

We also found that different TFs could have an additive effect on H3K27me3 marking. PcG proteins in plants seem to be involved in both a transient and long-term repression, the latter persisting through multiple cell divisions. Long-term repression has been reported to require the spreading and maintenance of high levels of H3K27me3 marks across the target genes (Costa and Dean, 2019). Interestingly, the initial repression of *FLC* requires the RY element for PcG recruitment/nucleation (Costa and Dean, 2019), which is the binding motif of VAL TFs, but its long-term repression involves other cis-regulatory sequences located along the *FLC* locus (Qüesta et al., 2020). Similarly, PcG-mediated long-term repression in Drosophila requires sequence-specific targeting of PRC2 (Laprell et al., 2017). Thus, it is possible that the combined action of different recruiting factors propagates and maintains the appropriate H3K27me3 levels to mediate long-term gene repression in Arabidopsis.

## Materials and methods

### Plant material and culture conditions

Seeds of *A. thaliana* Col-0 WT, *bmi1 abc* (Yang et al., 2013a), and transgenic plants harboring the different constructs were surface-sterilized in 10% (v/v) bleach for 20 min and washed four times with sterile double-distilled water. Sterilized seeds were cold stratified in the dark for 2–4 days at 4°C and then grown under long-day conditions (consisting of 16-h light using white fluorescent bulbs with a fluence rate of 70 μmol·m^−2^·s^−1^ and 8-h dark) at 21°C on MS agar plates containing 1.5% sucrose and 0.8% agar for 7 days. MS agar plates were appropriately supplemented with Kanamycin (50 µg·mL^−1^) and/or hygromycin (10 µg·mL^−1^) for the selection of transgenic plants.

### Synthetic system constructs and transgenic plants

To generate the synthetic target gene constructs, we used the *pCAMBIA 1305.1* binary vector containing the *GUS* reporter gene under the control of the *cauliflower mosaic virus* (*CaMV35S*) promoter as a backbone (Abcam; ab275762). We replaced the *CaMV35S* promoter by a *CaMV35S* in which the LexA DNA binding element (Lex A operator (*LexO*)) amplified from the *pER8* vector (Zuo et al., 2000) was cloned upstream of the *TATA* box into an *EcoR*V site as a blunt fragment, resulting in the *pLexO:GUS* construct. To generate the *p(G + 2T)LexO:GUS* construct, a DNA fragment of 100 bp containing one *GAGA* and two *TELOBOX* motifs was cloned upstream of the *LexO* into the reconstituted *EcoR*V site of the *pLexO:GUS* construct (see Supplemental Figure S10). This fragment was amplified from the regulatory region of *ABI3*. These constructs were transformed by the floral dip method (Clough and Bent, 1998) into WT Col-0 plants to generate WT/*pLexO:GUS* and WT/*p(G + 2T)LexO:GUS* transgenic plants. To build the BD translational fusion constructs, we inserted the *G10-90* promoter and the *LexA BD* (252-bp N-terminal region of *LexA*) amplified as an *EcoR*I-*Sac*I fragment from the *pER8* vector (Zuo et al., 2000), cDNA of the desired TF as a *Sac*I-*Sma*I fragment, and the *OCTOPINE SYNTHASE* terminator as a *BamH*I-*Pst*I fragment into the *pPZP211* vector (Hajdukiewicz et al., 1994) using the corresponding restriction sites. To construct the *BD–EAR* fusion, we used the C-terminal region of *VAL1 cDNA* containing a predicted nuclear localization signal (NLS) and the EAR domain (region from 2,041 bp to the stop codon of *VAL1 cDNA*; see Supplemental Figure S18). This fragment was inserted into the *Sac*I and *Sma*I sites of the modified pPZP211 vector. To ensure that the BD was transported to the nucleus when expressed alone, the sequence corresponding to the predicted NLS of VAL1 (region from 2,041 to 2,183 bp of *VAL1 cDNA*; see Supplemental Figure S18) was fused to the C-terminal region of the *BD* using *Sac*I and *Sma*I sites as before. The different BD fusion constructs were transformed into WT Col-0 plants. The expression of the protein in the different transgenic lines was verified by immunoblotting using anti-LexA BD antibody. The BD fusion lines were crossed to WT/*pLexO:GUS_1*, WT/*pLexO:GUS_2*, or WT/*p(G + 2T)LexO:GUS* plants, as indicated in the Results. Primers used to generate the different constructs are listed in Supplemental Data Set S3.

### Histochemical staining of GUS activity

GUS staining of transgenic plants was performed as described before (Calonje et al., 2008). Briefly, the tissue was incubated in 2-mM 5-bromo-4-chloro-3-indolyl-β-d-glucuronic acid in 50-mM phosphate buffer, pH 7.0, containing 0.5-mM K_3_Fe(CN)_6_ and 0.5-mM K_4_Fe(CN)_6_ for 2 h at 37°C. The tissue was then rinsed with 50-mM phosphate buffer and fixed in ethanol (95%):acetic acid (9:1, v/v) for 2 to 4 h at room temperature. Images were captured under an Olympus SZ40 Stereo Microscope.

### Immunoblot assay

Total proteins extracted from 10 seedlings at 10 days after germination were separated on a 10% sodium dodecyl sulfate polyacrylamide gel electrophoresis (SDS*–*PAGE) gel and transferred to a polyvinylidene difluoride (PVDF) membrane (Immobilon-P Transfer membrane, Millipore) by semi-dry blotting in 25-mM Tris–HCl, 192-mM glycine, and 10% methanol. To detect the fusion proteins, anti-LexA BD antibody (Millipore 06-719; 1:2,000) was used as the primary antibody and Horseradish peroxidase-conjugated goat anti-rabbit (Sigma-Aldrich, A0545; 1:10,000) as the secondary antibody. Chemiluminescence detection was performed with ECL Prime Western Blotting Detection Reagent (GE Healthcare Life Sciences) following the manufacturer’s instructions.

### Fluorometric assay of GUS activity

The activity of GUS was determined in whole seedlings using 4-methylumbelliferyl β-d-glucuronide (4-MUG) as a substrate (Halder and Kombrink, 2015). A single 7-day-old seedling was placed in each well of a 96-well microplate and incubated in 150-μL lysis buffer (50-mM sodium phosphate, pH 7.0, 10-mM EDTA, 0.1% Triton X-100) containing 1-mM 4-MUG at 37°C for 90 min. At the end of the incubation period, 50 μL of 1M Na_2_CO_3_ (stop solution) was added to each well, and 4-MU fluorescence was directly measured in a microplate reader (excitation/emission wavelength of 365/455 nm). Activity is expressed as relative fluorescence units.

### Gene expression analysis

Total RNA was extracted from 10 seedlings at 10 days after germination (DAG) using an RNeasy Plant Mini Kit (Qiagen), and on-column DNase treatment (Qiagen) was performed to remove any DNA contamination. cDNAs were reverse-transcribed from total RNAs with a QuantiTect Reverse Transcription Kit (Qiagen). For reverse transcription polymerase chain reaction analyses, amplifications were performed using a SensiFAST SYBR & Fluorescein Kit (Bioline) and the iQ5 Bio-Rad system. The program was as follows: melting at 95°C for 2 min and amplification with 40 cycles of 95°C for 5 s, 60°C for 10 s and 72°C for 10 s. *ACTIN2* was used as endogenous control. Primers used are specified in Supplemental Data Set S3.

### ChIP and ChIP-qPCR

ChIP experiments were performed on 1 g of 7-day-old seedling tissue fixed in 1% formaldehyde. Chromatin was extracted from the fixed tissue and fragmented using a Bioruptor Pico (Diagenode) in fragments of 200–500 bp. The sheared chromatin was immunoprecipitated overnight using the following antibodies and dilutions: anti-LexA BD (Millipore 06-719, 1:300), anti-H3K27me3 (Millipore, 07-449, 1:300), anti-H2AK121ub (Cell Signaling, 8240S; 1:100), anti-Histone H3 (acetyl K9 + K14 + K18 + K23 + K27) (Abcam ab47915, 1:300), or anti-H3K4me3 (Diagenode, C15410003-50; 1:300). Immunocomplexes were captured using Protein-A Sepharose beads CL-4B (GE Healthcare). After washing the Protein-A beads, chromatin was eluted and the cross-linking was reversed overnight at 65°C. The DNA from the immunoprecipitated chromatin was treated with RNase and proteinase K and purified by phenol–chloroform extraction followed by ethanol precipitation. For ChIP-qPCR, amplification was performed using a SensiFAST SYBR & Fluorescein Kit (Bioline) and the iQ5 BioRad system. Results are given as percentage of input or normalized to the levels of histone marks at *FLC*, *ACTIN7 (ACT7)*, or *AG*, depending on the histone mark and the genotype analyzed, as indicated in the Results section. qPCR data are shown as the means of two to three independent pools of tissue as indicated. Primers used for ChIP-qPCR are listed in Supplemental Data Set S3.

### Pull-down assay

For the in vitro pull-down assays, Arabidopsis *BMI1A* cDNA epitope tagged with *HA* was cloned into *pET19b* (Novagen), and *Nt-VAL1* and *Ct-VAL1* fragments were cloned into *pGEX-4T-3* (GE Healthcare) and transformed into *Escherichia coli* strain BL21 Rosetta. Primers used to build these constructs are listed in Supplemental Data Set S3. Cells expressing GST, GST fusion proteins, or BMI1A-HA protein were collected by centrifugation, re-suspended in 1 mL of extraction buffer (20-mM Tris–HCl, pH 7.5, 150-mM NaCl, 1-mM MgCl_2_, 0.1% Triton X-100, 10% glycerol, and 1 mM PMSF), and sonicated. The extracts were centrifuged for 20 min at 14,000 rpm at 4°C. An equal volume (300 µL) of GST or GST fusion protein extract was mixed with BMI1A-HA extract. For the pull-down assays, 30 μL of Glutathione-Sepharose beads (GE Healthcare) were added to the mixture and incubated for 2 h at 4°C. The beads were washed three times with Extraction Buffer. Standard procedures were used for immunoblotting using polyclonal anti-HA antibody (Sigma) (1:5,000).

### Statistical analysis

The data presented in the figures are reported as the sample mean ± sd. The statistical significance of the pairwise comparisons between the means of different samples was examined using a two-sided Student’s *t* test. The difference between the sample means was considered statistically significant with a *P <*0.05 (marked with a single asterisk), 0.01 (marked with two asterisks), or 0.001 (marked with three asterisks). This statistical analysis was performed using Microsoft Excel 2016 software.

### Accession numbers

Sequence data from this article can be found in the GenBank/EMBL libraries under the following accession numbers: VAL1 (AT2G30470); BMI1A (AT2G30580); RING1B (AT1G03770); KNU (AT5G14010); FLC (AT5G10140); ERF10 (AT1G03800); EMF1 (AT5G11530); ACT2 (AT3G18780); AG (AT4G18960); ACT7 (AT5G09810); ABI3 (AT3G24650).

Previously generated H3K27me3 and H2AK121ub ChIP-seq data are available in the Gene Expression Omnibus (GEO) under accession number GSE89358 and H3ac ChIP-seg data under accession number GSM4952436.

## Supplemental data

The following materials are available in the online version of this article.

**Supplemental Figure S1.** GUS activity and *GUS* expression in WT/*pLexO:GUS* lines.

**Supplemental Figure S2.** LexA BD fusion proteins.

**Supplemental Figure S3.** Levels of PcG marks at the *pLexO:GUS_2* reporter locus in plants expressing different BD fusion proteins.

**Supplemental Figure S4.** Expression of genes used as ChIP internal controls in different transgenic lines.

**Supplemental Figure S5.** ChIP results of Figure 2 showing normalized data to an internal control.

**Supplemental Figure S6.** *GUS* expression levels in transgenic lines in the presence of different BD fusion proteins.

**Supplemental Figure S7.** The interaction of VAL1 and BMI1 requires the C-terminal half of VAL1.

**Supplemental Figure S8.** Expression of BD-KNU, BD-FLC, and BD-ERF10 proteins.

**Supplemental Figure S9.** ChIP results of Figure 3 showing normalized data to an internal control.

**Supplemental Figure S10.** Motifs enriched at the 500-bp regions upstream of the ATG of only-H3K27me3 marked genes.

**Supplemental Figure S11.** Fragment used to build the *p(G + 2T)LexO:GUS* construct.

**Supplemental Figure S12.** GUS activity assay of the WT/*pLexO:GUS* and WT/*p(G + 2T)LexO:GUS* T1 lines.

**Supplemental Figure S13.** BD-KNU and BD-KNU(-EAR) proteins.

**Supplemental Figure S14.** ChIP results of Figure 4, B and C showing normalized data to an internal control.

**Supplemental Figure S15.** ChIP results of Figure 4, E, F, and H showing normalized data to an internal control.

**Supplemental Figure S16.** ChIP results of Figure 5 showing normalized data to an internal control.

**Supplemental Figure S17.** Expression of BD-EMF1 protein.

**Supplemental Figure S18.** VAL1 fragments used to build the BD and BD-EAR fusion proteins.

**Supplemental Data Set S1.** 6-mer motif analysis at the 500-bp region upstream of the ATG of PcG targets.

**Supplemental Data Set S2.** Coimmunoprecipitation/mass spectrometry (CoIP–MS) analysis using emf1-2/pEMF1:EMF1-3XFLAG complemented mutants.

**Supplemental Data Set S3.** Primers used in this study.

## Funding

This work was supported by grants from the Spanish Ministry of Science and Innovation (grant nos. BIO2016-76457-P and PID2019-106664GB-I00). I.H. was supported by a Spanish National Research Council (Consejo Superior de Investigaciones Científicas (CSIC)) training scholarship (JAEINT2018-EX-0821).

*Conflict of Interest statement*. The authors declare that they have no competing interests.

## Supplementary Material

koab139_Supplementary_Data

## Notes

F.B. and W.M. performed the experiments with the help of IH. M.C. designed the study, interpreted the results, and wrote the manuscript.

The author responsible for distribution of materials presented in this article in accordance with the policy described in the Instructions for Authors (https://academic.oup.com/plcell) is: Myriam Calonje (myriam.calonje@ibvf.csic.es).
