# Supplementary material for: EAR domain-containing transcription factors trigger PRC2-mediated chromatin marking in Arabidopsis
Source: Plant Cell. 2021 May 18;33(8):2701–15. doi: 10.1093/plcell/koab139 (PMC8408475; doi:10.1093/plcell/koab139)
Supplement: koab139_Supplementary_Data [file koab139_supplementary_data.zip › tpc.00689.2020-s01.pdf]

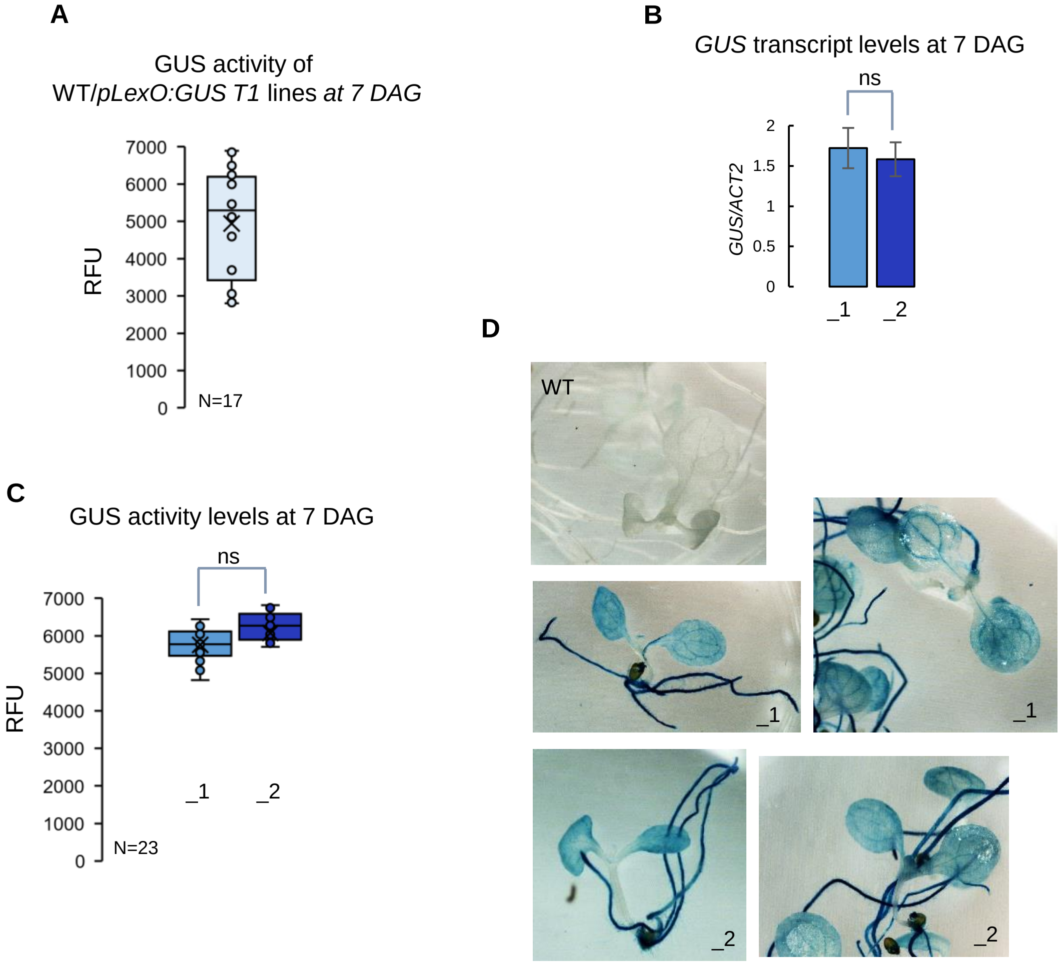

Supplemental Figure S1. GUS activity and *GUS* expression in WT/*pLexO:GUS* lines (Supports Figure 1). **(A)** Box plot showing GUS activity of different WT/*pLexO:GUS* T1 lines. Activity is expressed as relative fluorescence units (RFU). The median (segment inside rectangle), the mean (cross inside the rectangle), upper and lower quartiles (boxes), and minimum and maximum values (whiskers) are indicated. **(B)** qRT-PCR analysis of *GUS* reporter expression in WT/*pLexO:GUS_1 and _2* seedlings. Quantifications were normalized to *ACTIN2* (*ACT2*). Bars indicate standard deviation of two independent pools of tissue. No significant differences were found according to two-sided Student's t-test. **(C)** Box plots showing GUS activity of different WT/*pLexO:GUS_1 and _2* seedlings determined as in (A). Results from two independent experiments were combined. No significant differences were found according to two-sided Student's t-test. **(D)** GUS staining of WT, WT/*pLexO:GUS_1* and *_2* seedlings at 7 DAG. Blue staining indicates reporter GUS activity.

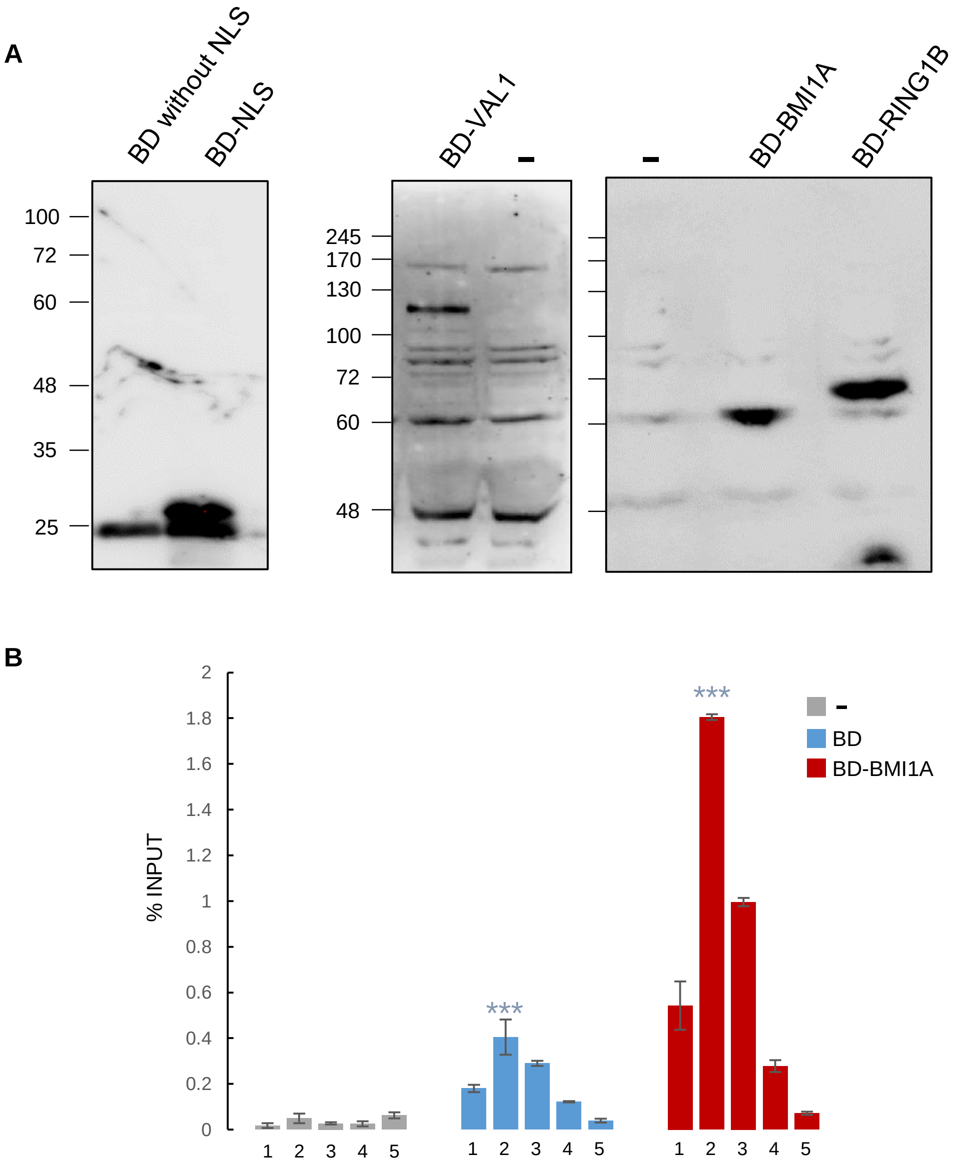

Supplemental Figure S2. LexA BD fusion proteins (Supports Figure 1). **(A)** Detection of BD fused to the Nuclear Localization Signal (BD-NLS), BD-VAL1, BD-BMI1A and BD-RING1B protein expression in the different WT transgenic lines at 10 DAG by immunoblotting analysis using anti-LexA BD antibody. Results confirmed the overexpression of all fusion proteins. **(B)** Bar charts showing BD and BD-BMI1A enrichment at *pLexO:GUS_2* reporter locus determined by ChIP using anti-LexA BD antibody. *WT/pLexO:GUS_2* plants were used as negative control (-). Numbers at x-axis indicate the position of amplified regions as indicated in Figure 1B. Results are indicated as percentage of input. Error bars represent standard deviation of n=2 independent pools of tissue. Significant differences compared to negative control as determined by two-sided Student's t-test are indicated (***P < 0.001).

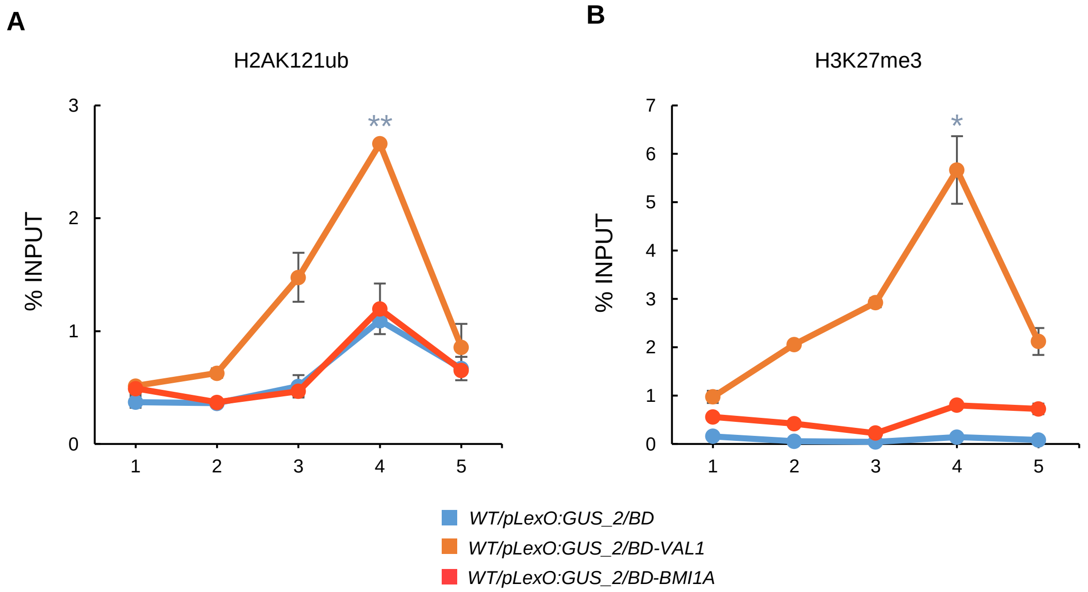

Supplemental Figure S3. Levels of PcG marks at *pLexO:GUS_2* reporter locus in plants expressing different BD fusion proteins (Supports Figure 2). **(A)** H2AK121ub levels at *GUS* reporter locus after the binding of *BD*, *BD-VAL1* or *BD-BMI1A*. **(B)** H3K27me3 levels at *GUS* reporter locus after the binding of *BD*, *BD-VAL1* or *BD-BMI1A*. Results show the recovery of immunoprecipitated material as percentage of input. Numbers at *x*-axis indicate the position of amplified regions as indicated in Figure 1B. Error bars indicate standard deviation of n=2 independent pools of tissue. Significant differences at position 4 are indicated as determined by two-sided Student's t-test (**P < 0.01; *P < 0.05).

Supplemental Data. Baile, Merini et al. (2021). EAR domain-containing transcription factors trigger PRC2-mediated chromatin marking in Arabidopsis. Plant Cell.

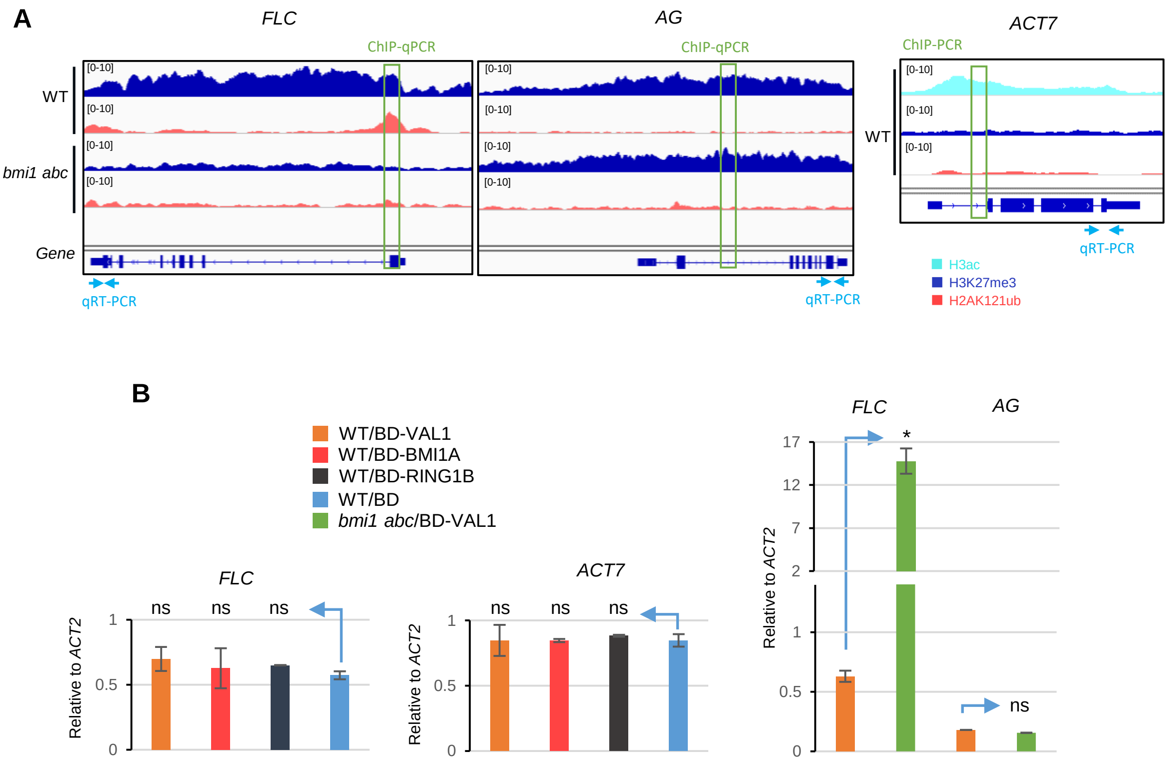

Supplemental Figure S4. Expression of genes used as ChIP internal control in different transgenic lines (Supports Figure 2). **(A)** Screenshots showing the levels of H3K27me3 and H2AK121ub at *FLC* and *AG* loci in WT and *bmi1 abc* mutants determined by ChIP-seq, and the levels of H3K27me3, H2AK121ub and H3ac at *ACT7* in WT. Notice that, unlike at *FLC*, the levels of H3K27me3 at *AG* are not significantly altered in *bmi1 abc* compared to WT. Regions amplified in ChIP-qPCR and qRT-PCR experiments are indicated. **(B)** qRT-PCR analysis of *FLC* and *ACT7* expression levels in WT/*pLexO:GUS_1/BD-VAL1,* WT/*pLexO:GUS_1/BD-BMI1A,* WT/*pLexO:GUS_1/BD-RING1B* and WT/*pLexO:GUS_1/BD*, and *FLC* and *AG* expression levels in WT/*pLexO:GUS_1/BD-VAL1* and *bmi1 abc*/*pLexO:GUS_1/BD-VAL1* seedlings at 10 DAG. Quantifications were normalized to *ACTIN2* (*ACT2*). Bars indicate standard deviation of two independent replicates. Significant differences are indicated as determined by two-sided Student's t-test (*P < 0.05; “ns” not significant).

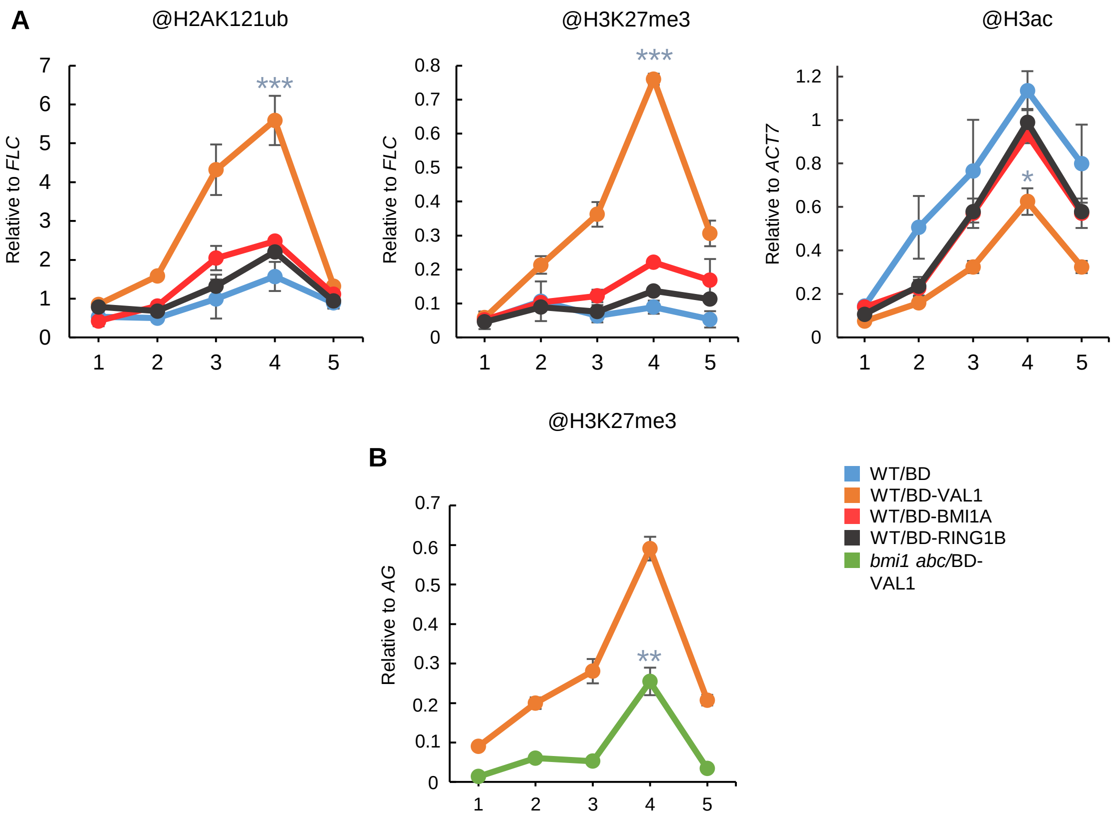

Supplemental Figure S5. ChIP results of Figure 2 showing normalized data to an internal control. (A) H2AK121ub, H3K27me3 and H3ac levels at *GUS* reporter locus in WT/*pLexO:GUS_1/BD*, WT/*pLexO:GUS_1/BD-VAL1,* WT/*pLexO:GUS_1/BD-BMI1A* and WT/*pLexO:GUS_1/BD-RING1B* plants. H2AK121ub and H3K27me3 levels were normalized to the levels at *FLC* and H3ac levels to the levels at *ACT7*. **(B)** H3K27me3 levels at *GUS* reporter *locus* in WT/*pLexO:GUS_1/BD-VAL1* and *bmi1 abc*/*pLexO:GUS_1/BD-VAL1* plants. H3K27me3 levels were normalized to the levels at *AG*. Numbers at *x*-axis indicate the position of amplified regions as indicated in Figure 1B. Error bars indicate standard deviation of n=2 independent pools of tissue. Significant differences at position 4 are indicated as determined by two-sided Student's t-test (***P < 0.001; **P < 0.01; *P < 0.05).

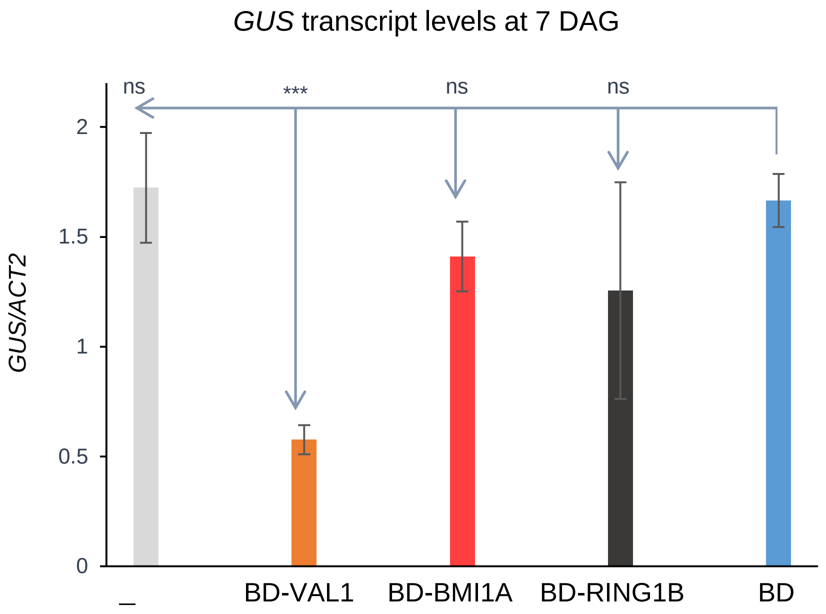

Supplemental Figure S6. *GUS* expression levels in transgenic lines in the presence of different BD fusion proteins (Supports Figure 2). qRT-PCR analysis of *GUS* reporter expression in WT/*pLexO:GUS_1*, WT/*pLexO:GUS_1/BD-VAL1*, WT/*pLexO:GUS_1/BD-BMI1A*, WT/*pLexO:GUS_1/BD-RING1B* and WT/*pLexO:GUS_1/BD* plants at 10 DAG. Quantifications were normalized to *ACTIN2* (*ACT2*). Bars indicate standard deviation of two independent pools of tissue. Significant differences between WT/*pLexO:GUS_1/BD* and the other plants determined by two-sided Student's t-test are indicated (***P < 0.001; “ns” not significant). *GUS* transcript levels in the different plants are in concordance with GUS activity levels.

Supplemental Data. Baile, Merini et al. (2021). EAR domain-containing transcription factors trigger PRC2-mediated chromatin marking in Arabidopsis. Plant Cell.

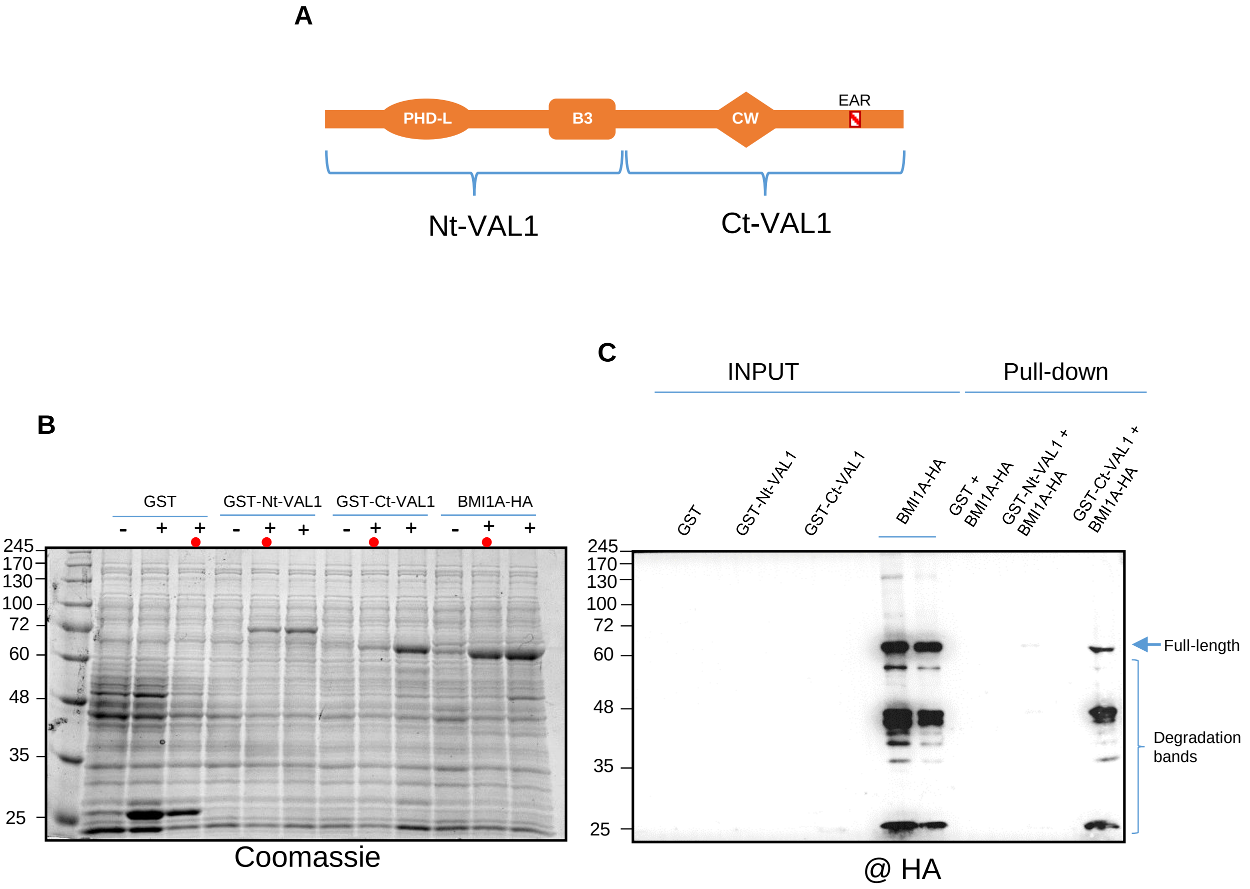

Supplemental Figure S7. The interaction of VAL1 and BMI1 requires the C-terminal half of VAL1 (Supports Figure 2). **(A)** Schematic representation of VAL1 in which the regions corresponding to Nt-VAL1 and Ct-VAL1 are indicated. **(B)** Coomassie gel staining showing the amount of GST, GST-Nt-VAL1, GST-Ct-VAL1 and BMI1A-HA proteins before (-) and after (+) IPTG induction. Red dots indicate the protein extracts used in pull-down assays. The Mw of protein markers is indicated. **(C)** Pull-down assay of BMI1A-HA, using GST or the different GST-tagged proteins. Pull-down fractions were analyzed by immunoblotting using anti-HA antibody. One representative blot of two independent experiments is shown.

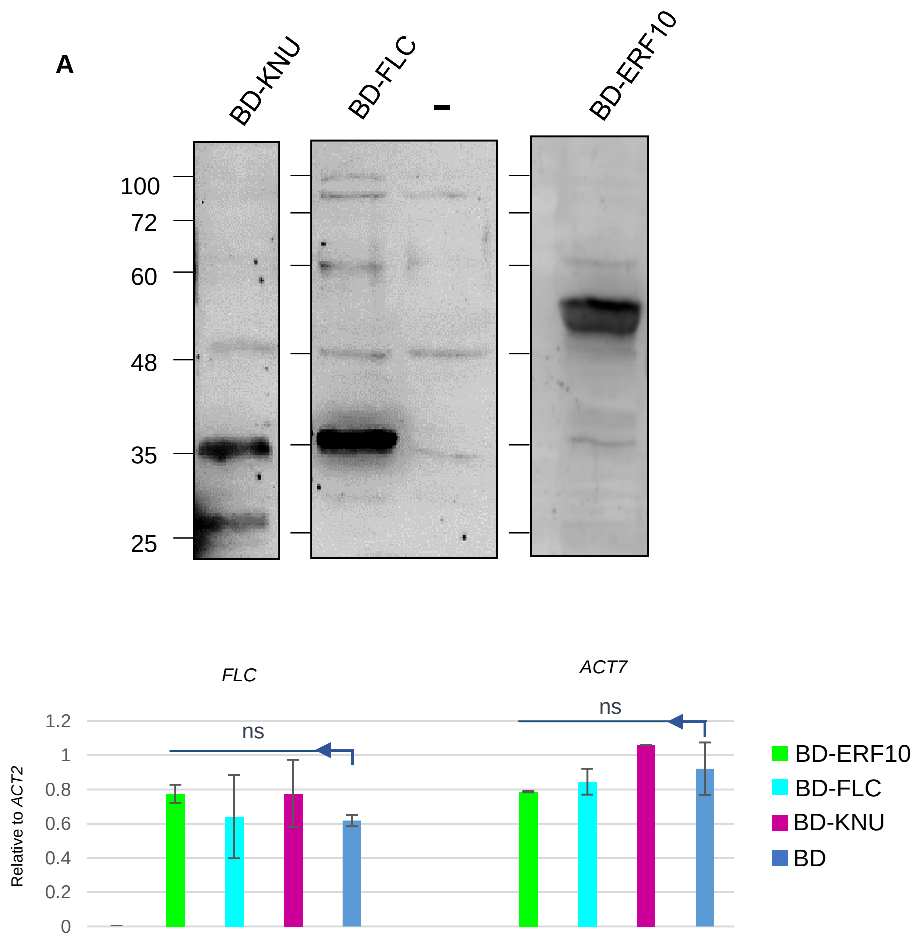

Supplemental Figure S8. Expression of BD-KNU, BD-FLC and BD-ERF10 proteins (Supports Figure 3). **(A)** Detection of BD-KNU, BD-FLC and BD-ERF10 in the different WT transgenic lines at 10 DAG by immunoblotting analysis using anti-LexA BD antibody. Results confirmed the overexpression of the fusion proteins. **(B)** qRT-PCR analysis of *FLC* and *ACT7* expression in WT/*pLexO:GUS_1/BD,* WT/*pLexO:GUS_1/BD-KNU,* WT/*pLexO:GUS_1/BD-FLC* and WT/*pLexO:GUS_1/BD-ERF10* seedlings at 10 DAG. Quantifications were normalized to *ACTIN2* (*ACT2*). Bars indicate standard deviation of two independent pools of tissue. Not significant (“ns”) differences as determined by two-sided Student's t-test were detected.

Supplemental Data. Baile, Merini et al. (2021). EAR domain-containing transcription factors trigger PRC2-mediated chromatin marking in Arabidopsis. Plant Cell.

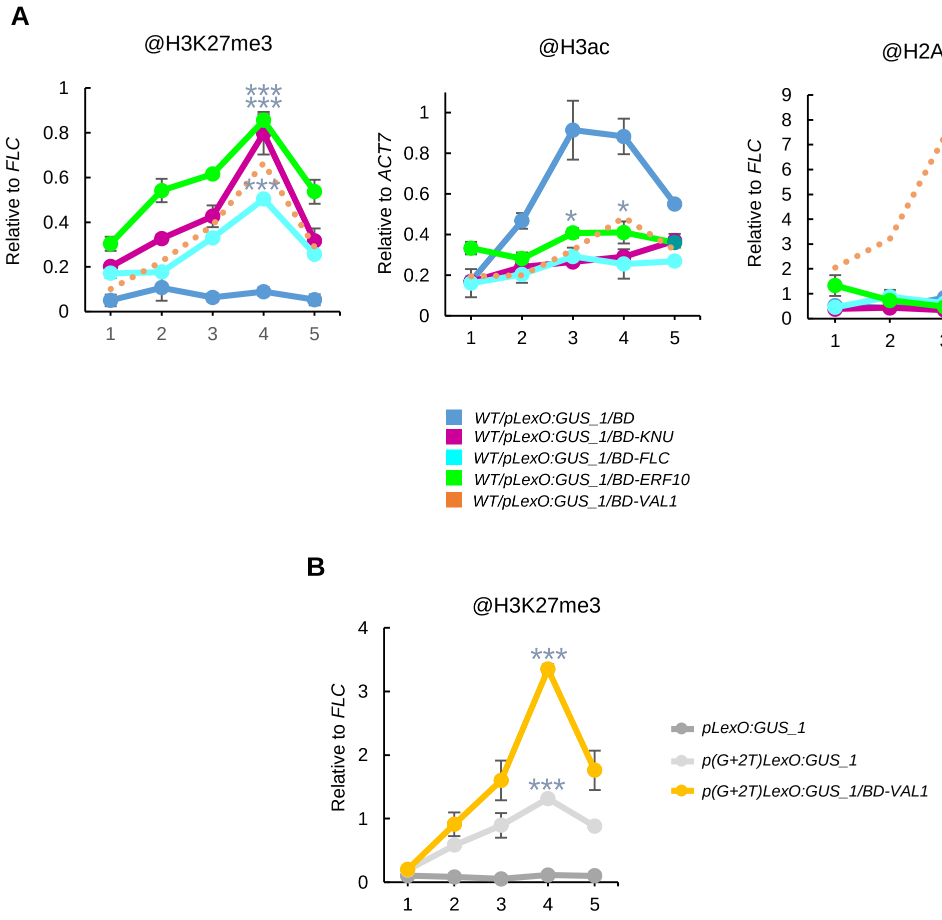

Supplemental Figure S9. ChIP results of Figure 3 showing normalized data to an internal control. (A) H3K27me3, H3ac and H2AK121ub levels at *GUS* reporter locus in WT/*pLexO:GUS_1/BD*, WT/*pLexO:GUS_1/BD-KNU,* WT/*pLexO:GUS_1/BD-FLC* and WT/*pLexO:GUS_1/BD-ERF10* plants. Results from one replicate of WT/*pLexO:GUS_1/BD-VAL1* plants were included as control (orange dotted line). H3K27me3 and H2AK121ub levels were normalized to the levels at *FLC* and H3ac levels to the levels at *ACT7*. **(B)** H3K27me3 levels at *GUS* reporter locus in WT/*pLexO:GUS_1* and WT/*p(G+T)LexO:GUS_1* and WT/*p(G+T)LexO:GUS_1/BD-VAL1* plants. H3K27me3 levels were normalized to the levels at *FLC.* Numbers at *x*-axis indicate the position of amplified regions as indicated in Figure 1B. Error bars indicate standard deviation of n=2 independent pools of tissue. Significant differences at position 4 compared to WT/*pLexO:GUS_1* determined by two-sided Student's t-test are indicated (***P < 0.001; **P < 0.01; *P < 0.05; “ns” not significant).

Supplemental Data. Baile, Merini et al. (2021). EAR domain-containing transcription factors trigger PRC2-mediated chromatin marking in Arabidopsis. Plant Cell.

Only-H3K27me3 genes

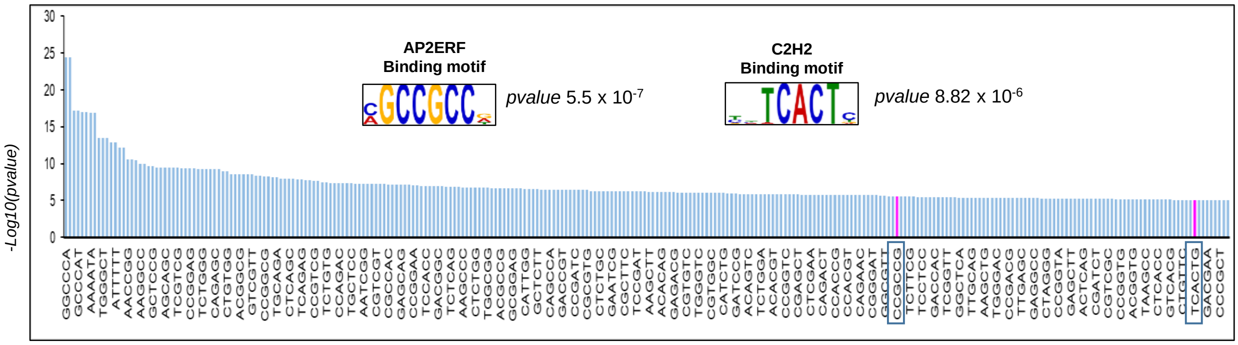

Supplemental Figure S10. Motifs enriched at 500 bp region upstream of the ATG of only-H3K27me3 marked genes (Supports Figure 3). Bar plot showing significantly enriched motifs at all only-H3K27me3 marked genes. Binding motifs of AP2ERF and C2H2 factors are indicated. Analysis was carried out using Tair Motif finder tool (https://www.arabidopsis.org/tools/bulk/motiffinder/index.jsp).

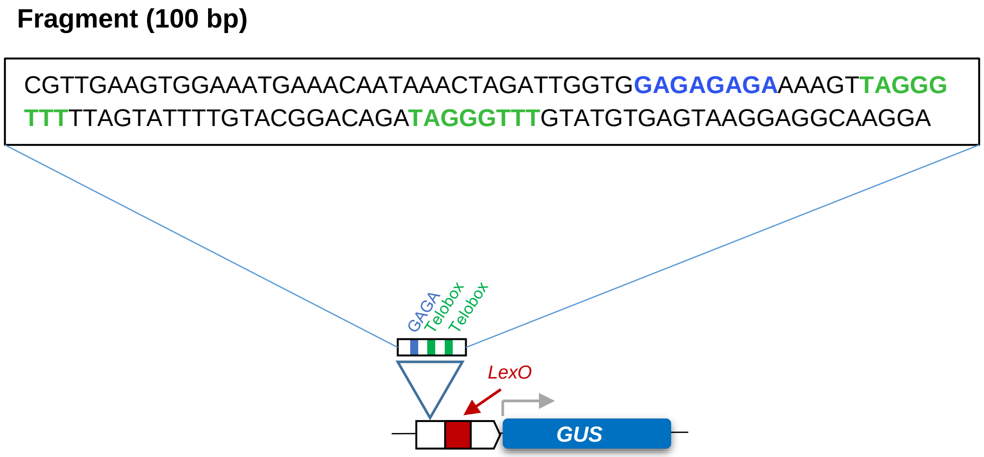

Supplemental Figure S11. Fragment used to build *p(G+2T)LexO:GUS* construct (Supports Figure 3). A fragment of 100 bp containing one *GAGA* and two *TELOBOX* motifs was cloned upstream of the *LexO.*

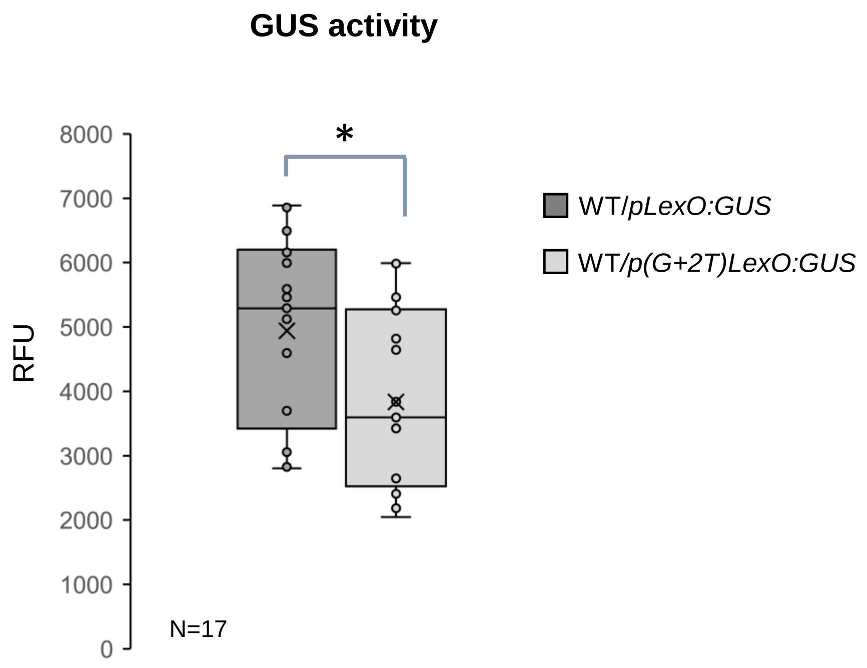

Supplemental Figure S12. GUS activity assay of WT/*pLexO:GUS* and WT/*p(G+2T)LexO:GUS* T1 lines (Supports Figure 3). Box plot showing differences in GUS activity among different T1 lines of the two type of transformants. Activity is expressed as relative fluorescence units (RFU). In each case, the median (segment inside rectangle), the mean (cross inside the rectangle), upper and lower quartiles (boxes), and minimum and maximum values (whiskers) are indicated. Significant differences as determined by two sided Student's t-test are indicated (*P < 0.05).

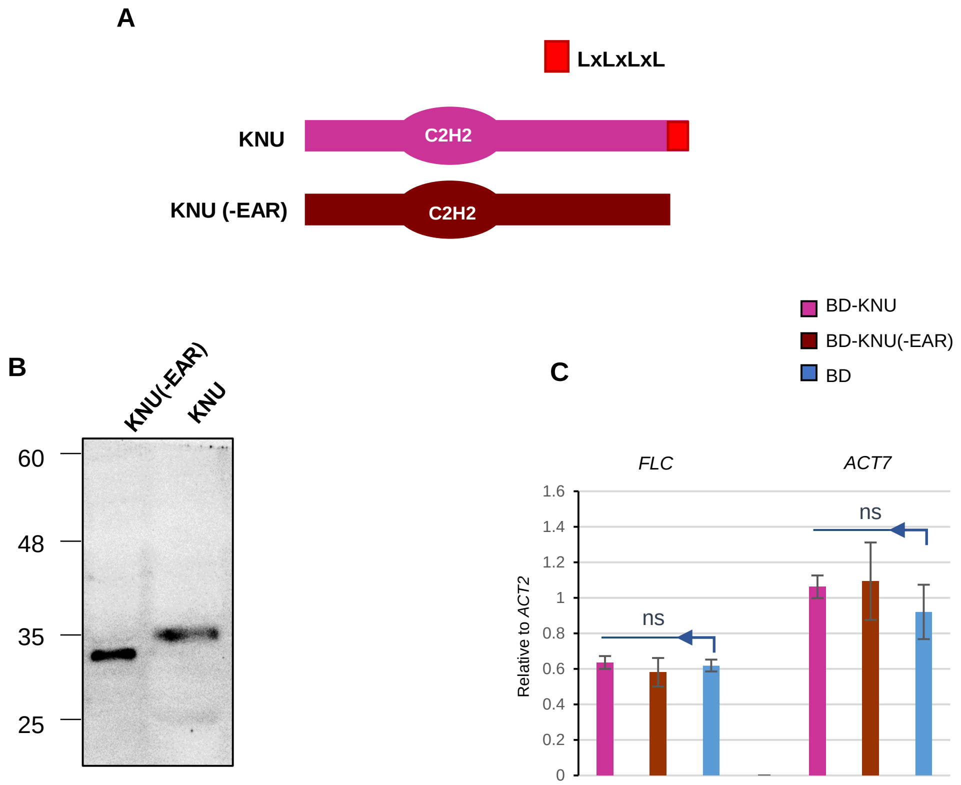

Supplemental Figure S13. BD-KNU and BD-KNU(-EAR) proteins (Supports Figure 4). **(A)** Schematic representation of KNU and KNU(-EAR). **(B)** Detection of BD-KNU and BD-KNU(-EAR) in transgenic lines at 10 DAG by immunoblotting analysis using anti-LexA BD antibody. Results confirmed the overexpression of the two fusion proteins. **(C)** qRT-PCR analysis of *FLC* and *ACT7* expression in WT/*pLexO:GUS_1/BD-KNU,* WT/*pLexO:GUS_1/BD-KNU(-EAR)* and WT/*pLexO:GUS/BD,* seedlings at 10 DAG. Quantifications were normalized to *ACTIN2* (*ACT2*). Bars indicate standard deviation of two independent pools of tissue. No significant (ns) differences were detected according to two-sided Student's t-test.

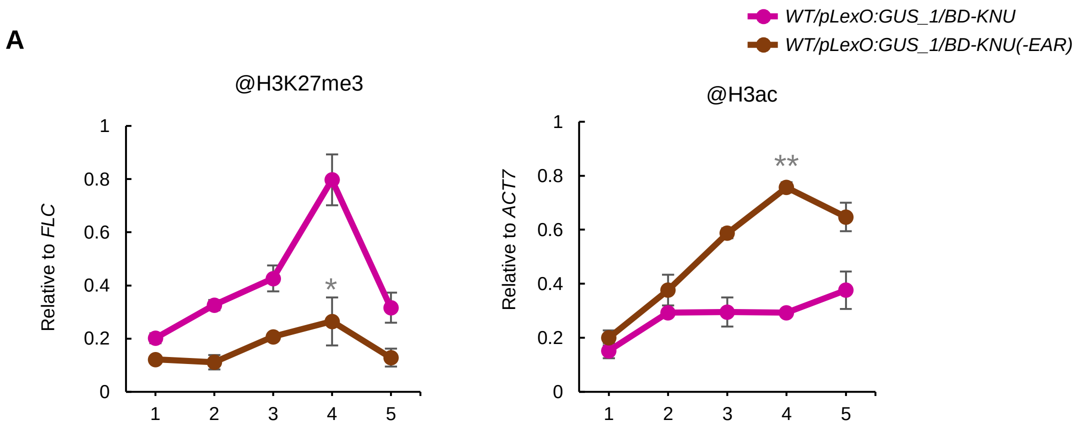

Supplemental Figure S14. ChIP results of Figure 4B and C showing normalized data to an internal control. H3K27me3 and H3ac levels at *GUS* reporter locus in WT/*pLexO:GUS_1/BD-KNU,* WT/*pLexO:GUS_1/BD-KNU(-EAR).* H3K27me3 levels were normalized to the levels at *FLC* and H3ac levels to the levels at *ACT7.* Numbers at *x*-axis indicate the position of amplified regions as indicated in Figure 1B. Error bars indicate standard deviation of n=2 independent pools of tissue. Significant differences between BD-KNU and BD-KNU(-EAR) at position 4 are indicated as determined by two-sided Student's t-test (**P < 0.01; *P < 0.05; “ns” not significant).

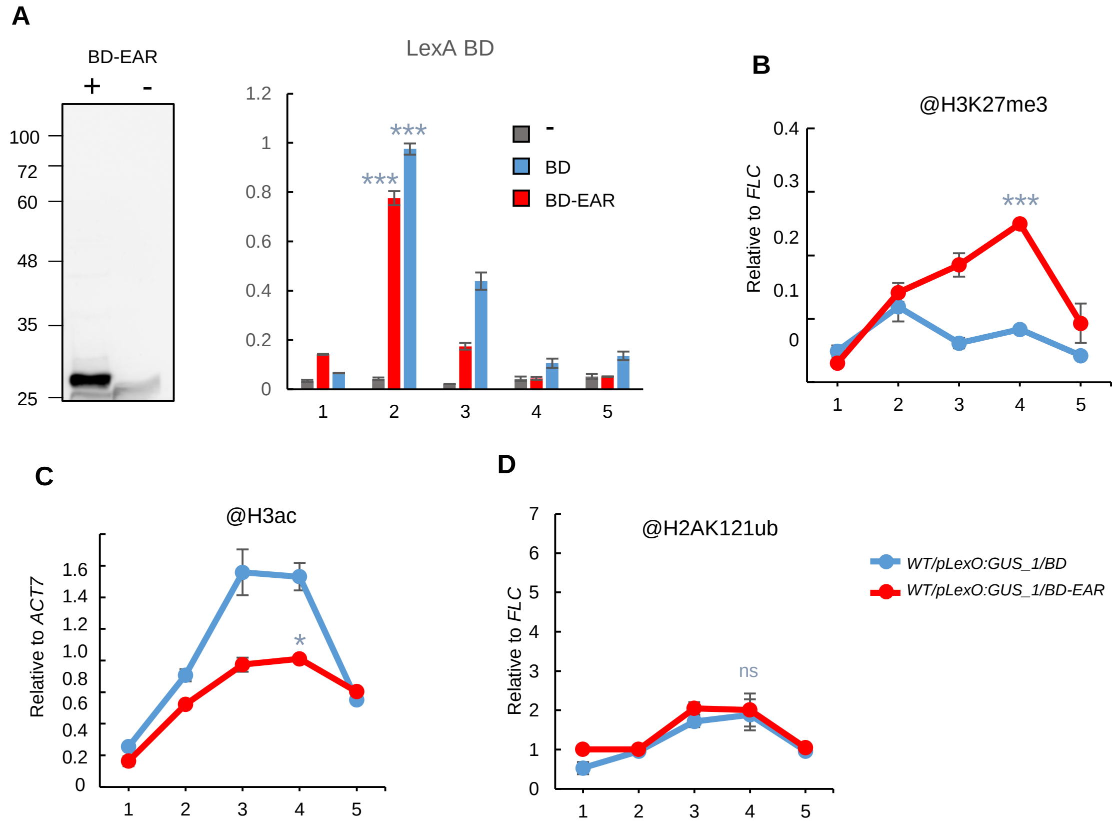

Supplemental Figure S15. ChIP results of Figure 4E, F and H showing normalized data to an internal control (Supports Figure 4). **(A)** Left panel, detection of BD-EAR in transgenic lines at 10 DAG by immunoblotting using anti-LexA BD antibody. Two lines, one expressing (+) and the other one not expressing (-) the protein are shown. Right panel, bar chart showing BD and DB-EAR enrichment at *pLexO:GUS_1* reporter locus determined by ChIP using anti-LexA DB antibody. WT/*pLexO:GUS_1* plants (-) were used as negative control. Results are indicated as percentage of input. Error bars represent standard deviation of n=2 independent pools of tissue. Significant differences at position 2 compared to control were determined by two-sided Student's t-test (***P < 0.001). Numbers at *x*-axis indicate the position of amplified regions as indicated in Figure 1B. **(B,C,D)** H3K27me3, H3ac and H2AK121ub levels at *GUS* reporter locus in WT/*pLexO:GUS_1/BD* and WT/*pLexO:GUS_1/BD-EAR*. H3K27me3 and H2AK121ub levels were normalized to the levels at *FLC* and H3ac levels to the levels at *ACT7.* Error bars indicate standard deviation of n=2 independent pools of tissue. Significant differences at position 4 are indicated as determined by two-sided Student's t-test (*P < 0.05; "ns" not significant).

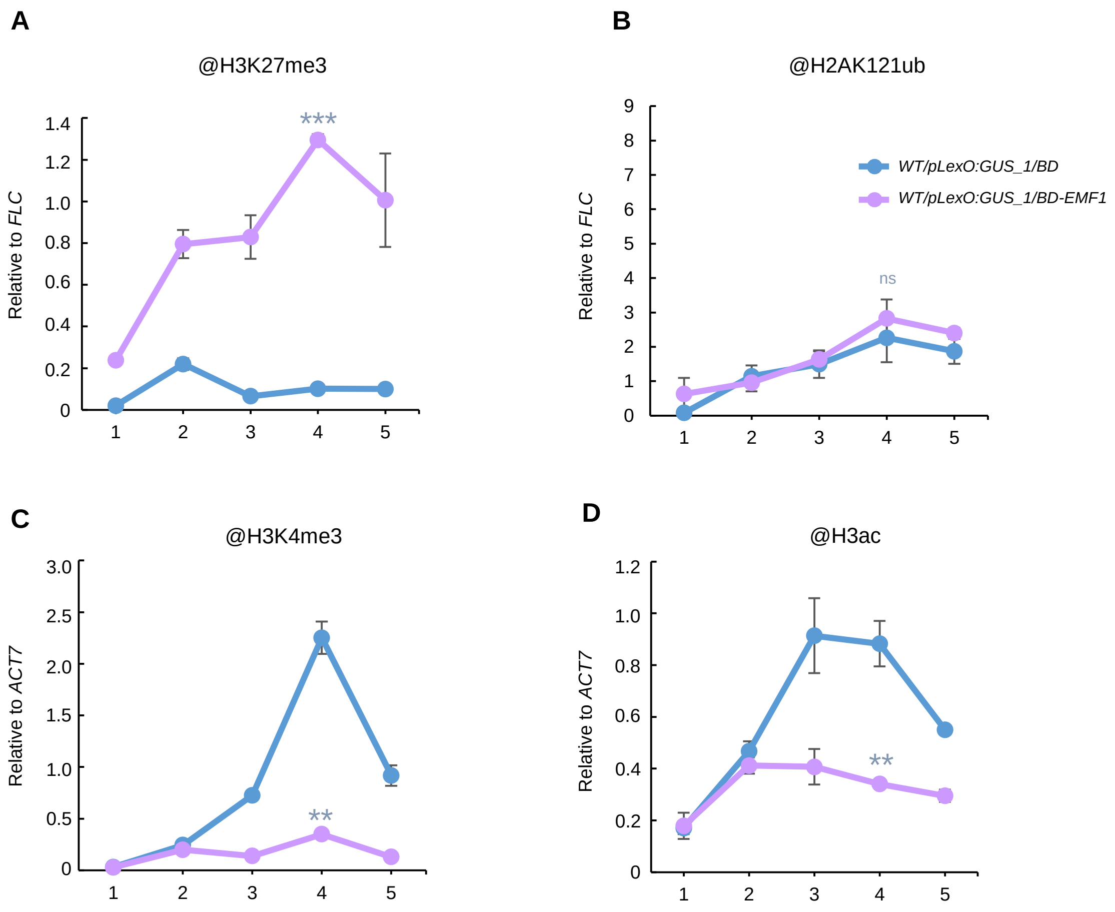

Supplemental Figure S16. ChIP results of Figure 5 showing normalized data to an internal control. (A,B,C,D) H3K27me3, H2AK121ub, H3K4me3 and H3ac levels at *GUS* reporter locus in WT/*pLexO:GUS_1/BD* and WT/*pLexO:GUS_1/BD-EMF1*. H3K27me3 and H2AK121ub levels were normalized to the levels at *FLC*, and H3K4me3 and H3ac levels to the levels at *ACT7*. Error bars indicate standard deviation of n=2-3 independent pools of tissue. Significant differences at position 4 are indicated as determined by two-sided Student's t-test (***P < 0.001; **P < 0.001; “ns” not significant).

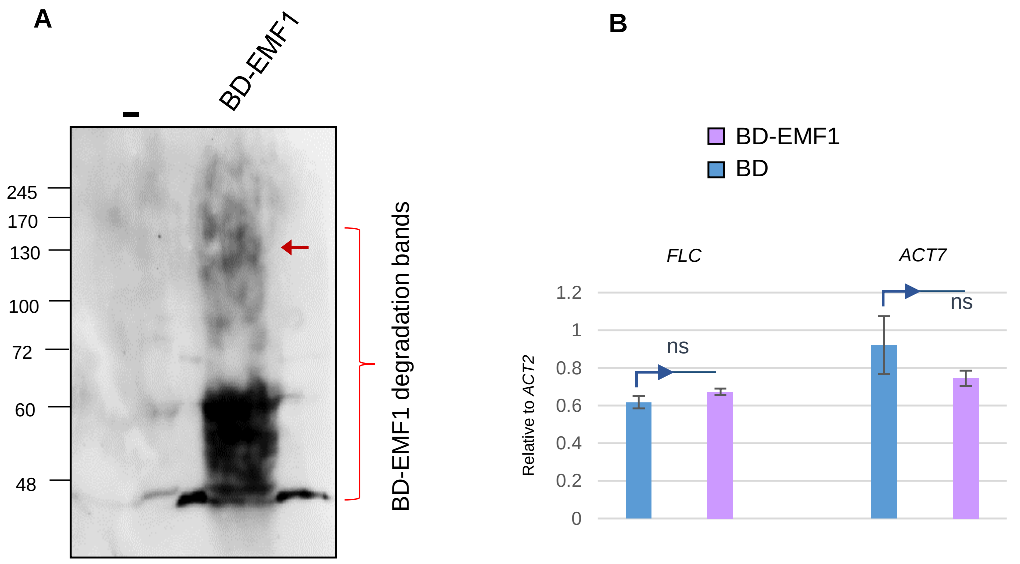

Supplemental Figure S17. Expression of BD-EMF1 protein (Supports Figure 5). **(A)** Detection of BD-EMF1 in transgenic seedlings at 10 DAG by immunoblotting analysis using anti-LexA BD antibody. BD-EMF1 is predicted to have a Mw of around 131 KDa; however, we could barely identify the full length protein. Instead, we observed several smeared bands with smaller Mw, indicating protein degradation. In any case, immunoblot results confirmed the overexpression of the fusion proteins. **(B)** qRT-PCR analysis of *FLC* and *ACT7* expression in WT/*pLexO:GUS_1/BD,* WT/*pLexO:GUS_1/BD-EMF1* seedlings at 10 DAG. Quantifications were normalized to *ACTIN2* (*ACT2*). Bars indicate standard deviation of two independent pools of tissue. Significant differences are indicated as determined by two sided Student's t-test (ns "not significant").

A

B

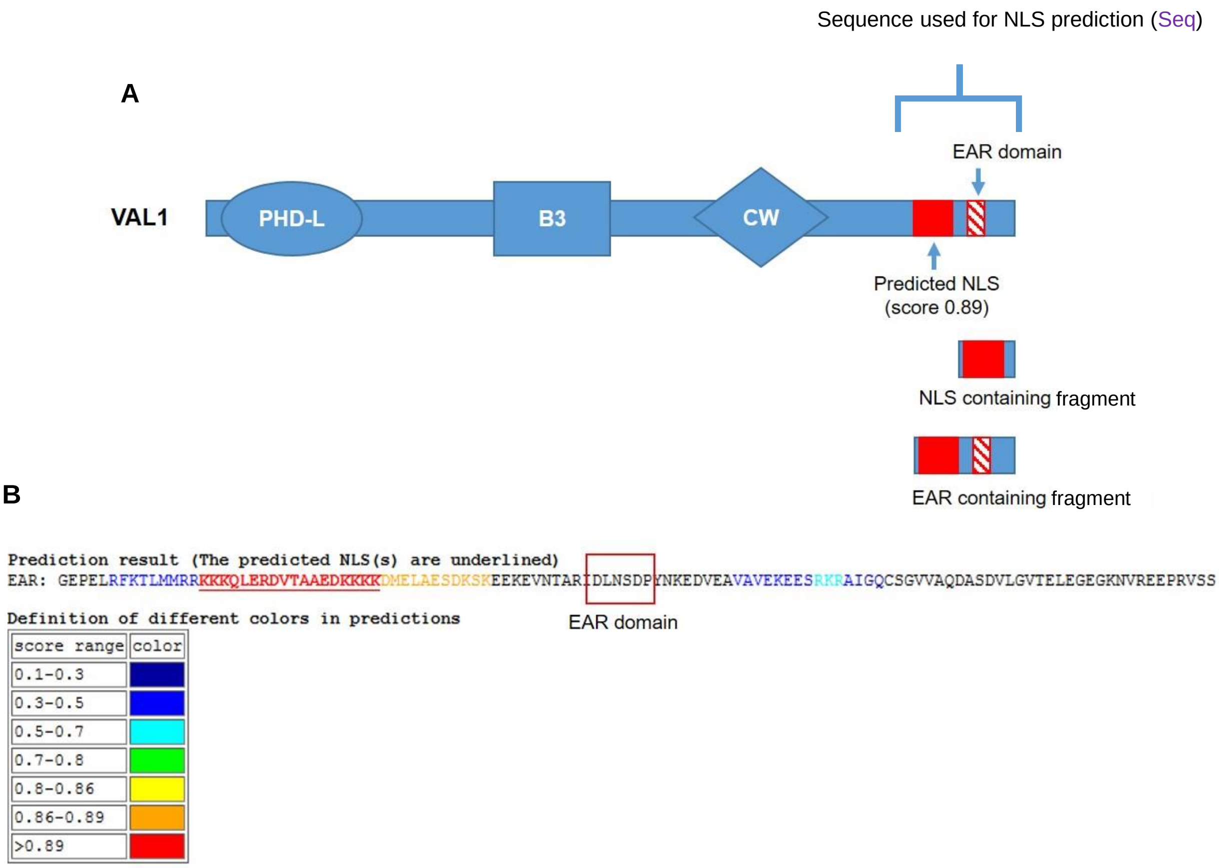

Supplemental Figure S18. VAL1 fragments used to build BD and BD-EAR fusion proteins (Supports Methods section). **(A)** Schematic representation of VAL1 showing the regions used to generate the different constructs. For the BD-EAR fusion, we used the C-terminal region of VAL1 that contains a predicted Nuclear Localization Signal (NLS) and the EAR domain. For BD alone, the sequence corresponding to VAL1 predicted NLS was fused to the C-terminal region of the BD to ensure that the BD was transported to the nuclei. **(B)** The predicted NLS with higher score according to SeqNLS tool (http://mleg.cse.sc.edu/seqNLS/) was used to generate these constructs.
