# Supplementary material for: EAR domain-containing transcription factors trigger PRC2-mediated chromatin marking in Arabidopsis
Source: Plant Cell. 2021 May 18;33(8):2701–15. doi: 10.1093/plcell/koab139 (PMC8408475; doi:10.1093/plcell/koab139)
Supplement: koab139_Supplementary_Data [file koab139_supplementary_data.zip › tpc.00689.2020-s06.pdf]

EAR domain-containing transcription factors trigger PRC2-mediated chromatin marking in Arabidopsis

Fernando Baile, Wiam Merini, Inés Hidalgo, Myriam Calonje

Corresponding author: Myriam Calonje myriam.calonje@ibvf.csic.es

Review timeline:

**TPC2020-RA-00689**	Submission received:	Aug. 28, 2020
	1st Decision:	Oct. 12, 2020 *Decline with external review*
**TPC2020-RA-00689R1**	1st Revision received:	Feb. 19, 2021
	2nd Decision:	March 19, 2021 *revision requested*
**TPC2020-RA-00689R2**	2nd Revision received:	Apr. 6, 2021
	3rd Decision:	May 4, 2021 *accept with minor revision*
**TPC2020-RA-00689R3**	3rd Revision received:	May 6, 2021
	4th Decision:	May 6, 2021 *acceptance pending, sent to science editor*
	Final acceptance:	May 14, 2021

REPORT: (The report shows the major requests for revision and author responses. Minor comments for revision and miscellaneous correspondence are not included. The original format may not be reflected in this compilation, but the reviewer comments and author responses are not edited, except to correct minor typographical or spelling errors that could be a source of ambiguity.)

TPC2020-RA-00689 1st Editorial decision – *decline with external review* Oct. 12, 2020

As you will see from the detailed comments of three reviewers, they find the topic of the work potentially very interesting. However, they all agree that the work has flaws in the experimental design and lacks important controls and independent replicates. Main concern are the single, not sufficiently characterized transgenic lines, the large differences in protein quantity that make comparisons difficult, and the inappropriate normalization procedures, in addition to several detailed points. Therefore, the reviewers did not find sufficient support for the conclusions drawn. As the problems are not easily eliminated without substantial new experimental work, we must decline the manuscript. Of course, this decision requires judgment and as working scientists who ourselves are subject to peer-review, we recognize that we may sometimes mis-judge important work or misinterpret its scope. We hope that this is not the case here and the comments will help to revise the work. If you decide to revise the experimental design and resubmit the work to The Plant Cell, it will be evaluated as a new submission subject to full assessment by the editorial board, including pre-review and editor selection, and if sent for external review, a new set of reviewers is likely to be chosen.

You may wish to consider publication in one of our sister journals, Plant Physiology and Plant Direct. You can transfer your manuscript and associated submission data automatically to these journals via the link below.

-- Reviewer comments:

Reviewer #1 (Comments for the Author):
The manuscript focuses in the PRC1 and PRC2 recruitment to their targeted genes. This complex process, where many players are involved, is clearly explained by the authors in the main text, addressing many aspects of this repression system. For that reason, it is highly appreciated that the authors make an up to date description of the known PRC1 and PRC2-recruiting proteins and DNA elements, emphasizing the missing parts needed to understand the recruitment mechanism. They address many of the unknown details of this process, specially completing the lack of information in the PRC1 and PRC2 interdependence and the role of TF to recruit them.

The authors show results that proves:
- Recruitment of PRC2 by VAL1 through PRC1.
- Recruitment of PRC2 independently of PRC1 by EAR domain-containing TFs.

- Synergistic effect in PRC2 recruitment by TFs.
- The effect of PRC2 (through EMF1) on other marks (H3K4me3 and H3ac).

Major revisions
1- The recruitment of the BD to the LexO promoter has been proved by performing an anti-LexA ChIP (Fig 1C and D). Are these panels representing one of the three independent transformed lines selected? From the text (line 139 and Suppl Fig1) and the Methods section, it is understood that three independent lines of WT/pLexO::GUS where crossed with one line with the BD domain. If the authors have tested by ChIP the BD binding with different lines, are the %Input in the BD-VAL1 lines always higher than the BD lines? In the Figure, BD-VAL1 ChIP results in approx. 5 times higher binding than BD. The authors do not address this difference in the text. The perfect negative control should have the same binding efficiency than BD-VAL1. I suggest the authors to address this point, preferentially by showing that other BD line (with higher binding efficiency) is not resulting in higher H2AK121ub/H3K27me3/H3ac.
2- The authors represent H2AK121ub and H3K27me3 levels referred to FLC and H3ac to ACT7, instead of %Input. The reason of this choice is not clarified in the text. This normalization has to be taken into consideration especially when analysing the levels of H3K27me3 in the atbmi1abc/pLexO::GUS/BD-VAL1 plants (Fig 2E). If FLC (or AG?) will be used for normalization, the authors should show that the levels of H3K27me3 in FLC are not affected in the mutant background, as it happens with most of the H2AK121ub/H3K27me3 marked genes in this mutant.
3- The authors show that many TFs, including VAL1, can recruit PRC2 through the EAR domain. To explore if the EAR domain is the point of recruitment of PRC2 and HDAC (through TPL/TPR or SAP18), they transformed plants with the pLexO::GUS/BD-KNU(-EAR) construct. They see a reduction of levels of H3K27me3 (Fig 4B), but also a low protein abundance compared to the other BD-KNU (Suppl Fig 2). Therefore, the reduction of H3K27me3 could be because a lower amount of the BD-KNU(-EAR), not due to the lack of activity in the absence of the EAR domain. Other TFs, or even VAL1 without the EAR domain could be interesting to test to reinforce this point. In fact, the BD-VAL1(-EAR) construct could be used also to address the point number 1, as it is expected to have the same binding efficiency than BD-VAL1 but should not be able to attract PRC2 activity.
The authors double verify the EAR domain role of attracting PRC2 by using a BD-EAR construct, but again there is a big difference when protein abundance is compared: BD-EAR amounts are many orders of magnitude higher than BD-KNU. This does not exclude the role of the EAR domain recruiting PRC2, but the higher efficiency of BD-KNU (that is able with lower amounts to induce higher levels of H3K27me3) could mean that other domains of the TF can also participate in the recruitment of PRC2.
4- The model proposed by the authors will be strengthened if the role of TPL/TPR and SAP18 connecting VAL1/TF with PRC2 could be tested using their in vivo system. One possibility to explore is to use the BD constructs in TPL/TPR or SAP18 defective mutant background.

Minor revisions
- Fig. 2E. It is indicated in the figure "relative to AG" and in the legend to FLC.
- Fig. 3. Colours of the lines in the panels C, D and E are very similar. Specially between BD and BD-FLC.
- Line 351. Reference marked as a number (16).

Reviewer #2 (Comments for the Author):
The paper by the Calonje lab aims to dissect PRC1 and PRC2 recruitment in plants. While some of the approaches are promising, it its current form the data provided does no support the conclusions drawn.

My main concerns are as follows

1. The authors anchor various constitutively overexpressed transcription factors to a synthetic regulatory element in the context of the 35S promoter and assess effects on GUS reporter expression and histone modifications. In this system, it is impossible to detect which events are directly caused by the factor of interest. Once Val1 recruits H2Aub and gene silencing occurs, this may secondarily lead to H3K37me3 and H3 deacetylation. This applies to all Figures. Although they repeat the Val1 recruitment in atbm1abc - they do not show what the H2AK121Ub or H3ac level is in the mutant background, precluding any conclusions about how these events are linked. The types of conclusions

drawn here can only be obtained with conditional gain-of-function TFs that allow a time course analysis to distinguish direct from indirect effects.
2. It seems from the methods, that the authors characterized only one transgenic line per transgene construct and that the data in the figures is derived from that line. We have no idea which if any constructs are functional. Moreover, the amount of the proteins present for the different transfactors varies widely (Fig. S2). KNU -EAR seems to be present at less than 10% of full-length KNU, which may explain the reduced H3K27me3 and increased H3K27ac in those plants relative to full-length KNU - rather than the lack of the EAR domain. On the other hand, some silencing tests rely on endogenous factors (GA and telobox experiment). There is no comparison at all possible for the effect of endogenous factor recruitment compared to the overexpressed transfactors. This experimental setup renders the small differences seen in many of the experiments meaningless unless they are normalized over protein levels or - independent transgenic lines are compared that have a similar expression levels.
3. To shore up the conclusion that "Dissection of PRC1 and PRC2 recruitment in Arabidopsis connects EAR repressome to PRC2 anchoring" one would expect the authors should have to tested physical interactions between the transfactors and PRC1 and/or HDAC components using full-length and various deletion constructs. Xiao et al. already identified interactions between PRC2 and TOE1 and AZF1 that are mediated by protein domains other than the EAR motif containing domain - in conflict with the model presented here.
4. Why did the authors use a piece from the *ABI3* 5'UTR to test GA repeat and telobox activity? The fragment they used is from the *ABI3* 5'UTR just upstream of the ATG and is cloned in the reverse orientation next to the LEXA binding site. This is very strange - no internal control is provided to assess how introducing this 100 bp piece of DNA alone affects the expression or histone marking of this new synthetic promoter, for example by mutating the GA and telobox motifs it contains. Thus, not only do some assays rely on overexpressed and other on endogenous trans factors, but the artificial promoter for the latter is entirely different form that used for the former. This precludes all comparisons. This paper is not about endogenous recruitment sites and cis motifs but about activity of transfactors. This could have been tested using AZF1 and BPC1 overexpressed in the same manner as all other transfactors tested here. Using lines with equal protein levels of each factor, one can then at least compare H3K27me3 levels caused (directly or indirectly) by factor tethering.
5. Not surprisingly, given the points above, there does not seem to be a linear relationship between H3K27me3 accumulation and reporter assays. In Fig. 2B VAL1 recruitment leads to ca. 0.8 units of H3K27me3 relative to FLC and a 50% reduction in GUS expression (Fig. 2D). In Fig. 3C, a very similar H3K27me3 level after recruitment of KNU, ERF10 over FLC instead leads to an 84% reduction in GUS expression. Finally, in Fig. 4 VAL1 recruitment causes 2 units of H3K27me3/FLC, and again only 50% reduction in GUS. p(G+2T), which aims to recruit endogenous BPC and Zn Finger proteins yields 1 unit of H3K27me3/FLC but only 20% reduction of reporter expression.

Additional minor points that need addressing

6. The changes in in histone modification should be reported relative to unmodified H3 or H2A or at a minimum as % input and not normalized over another loci (FLC, AG or ACT).

7. Why are the TF motifs displayed in Fig. 1, 2 and 3 although they are being tethered to the artificial promoter by LexA. This obfuscates the assay and does not add much novelty, many of these motifs had already been reported.

Reviewer #3 (Comments for the Author):
Polycomb-group proteins regulate thousands of genes in plants and thus contribute to numerous processes in plants. The two major Polycomb-repressive complexes PRC1 and PRC2, which catalyze H2AK121ub and H3K27me3, respectively, mediate this repression, and our understanding how these two PRCs are interacting and are recruited to their target genes is increasing. Recruitment is mediated at least in part by various transcriptions factors that confer a DNA-sequence specific binding of the complexes.

While the situation is very complex at individual genes, as many different binding sites are contained within a promoter/genomic environment, a system is lacking which separates the activity of the different recruiters on gene repression, PRC1 and PRC2 recruitment.

Baile et al. now developed a very interesting system which contains a synthetic promoter with a LexA binding site driving a GUS gene and LexA-TF fusions to dissect the function of the different TFs in gene silencing and PRC1/PRC2 recruitment. While the authors come up with many important conclusions (VAL1 recapitulates PRC1 and PRC2 marking, PRC2 tethering involves both VAL1 and PRC1, PRC2 can be recruited independently of PRC1, the EAR domain of the TFs is required for the recruitment), I have strong concerns about the presented data which needs clarification, further description and potentially further analyses. All the conclusions rely on comparability of the different lines (synthetic promoter, BD-lines) and it is unclear in the whole manuscript how these lines where analysed and established, particularly as the method section is very brief, and it remains unclear how many independent transgenic lines where analysed and how many replicates were performed. In addition, all the data are heavily normalized, which makes an analyses of the data difficult. Without all this information, a proper review is not possible and at this stage, most of the conclusions are not justified.

Major points:
1. e.g. line 141: it should be described what are the criteria to select "appropriate lines", are only single copy insertions identified, analysed and combined? How were the lines selected that are used throughout the manuscript? How many lines were independently analysed? This is also relevant for all the BD lines.
2. figures 1C/D: the %IP for BD-VAL1 is much higher than just BD, which may reflect differences in the lines (number of transgenes)? As these strong differences exist, this may also have an influence on H2AUbi and H3K27me3 recruitment. The analyses should be provided for at least two independent transgenic lines.
3. In addition, the different transcription factor lines show very different protein level (sup fig 2), and likely are therefore hardly comparable. For instance, BD-KNU (-EAR) is expressed at much lower levels than BD-KNU (sup fig 2). The authors show that BD KNU EAR represses much weaker than BD KNU (fig 4D) - how can the authors exclude that this is just a function of different protein level in the different constructs?
4. ChIP data are always normalized to *FLC* or *ACT7* - is the expression of these genes affected by the transgenes? Raw data (% of IP) should be shown for the ChIP experiments, so that it is possible to comment on the quality and comparability of the ChIP experiments.
5. LexA BD recruitment is to regions 1-3 (where the LexA binding site is) (fig 1) but histone marks are recruited to region 4 - (e.g. fig 2) - how is this explained? Is this a specific feature of the used GUS gene? Thus, further regions in the transgene (5´ of the promoter should be analysed and ideally transgenes without the GUS gene should be analysed.
6. GUS activity is shown as an average of different seedlings. It seems that only one replicate was performed - how many independent experiments were performed? Also stained seedlings should be shown to reveal that GUS expression is present in the same tissues.

Minor points:
line 121: the structure of this paragraph should be reordered, as it seems rather part of the introduction, but then includes some new data
in sup figure 1: the differences between the two different transgenics lines should be described in the legend as it is described in different parts of the main text

TPC2020-RA-00689R1 1st Revision received Feb. 19, 2021

Reviewer comments and **author responses**:

Reviewer #1:

Point 1. The recruitment of the BD to the LexO promoter has been proved by performing an anti-LexA ChIP (Fig 1C and D). Are these panels representing one of the three independent transformed lines selected? From the text (line 139 and Suppl Fig1) and the Methods section, it is understood that three independent lines of WT/pLexO::GUS

where crossed with one line with the BD domain. If the authors have tested by ChIP the BD binding with different lines, are the %Input in the BD-VAL1 lines always higher than the BD lines? In the Figure, BD-VAL1 ChIP results in approx. 5 times higher binding than BD. The authors do not address this difference in the text. The perfect negative control should have the same binding efficiency than BD-VAL1. I suggest the authors to address this point, preferentially by showing that other BD line (with higher binding efficiency) is not resulting in higher H2AK121ub/H3K27me3/H3ac.

RESPONSE: We apologize as we did not explain clearly the transgenic lines that were used in the previous version of the manuscript. This has been now clarified (see results section line 148-175, also see New Supplementary Figure 1). To ensure that the reporter locus was in the same chromatin environment in all cases, each BD overexpressing line was crossed to WT/pLexO::GUS_1 line. Nevertheless, to investigate possible variations in a different chromatin environment, some of these constructs were crossed to WT/pLexO::GUS_2 line (those results have been included in the new version also). We in addition generated a WT/p(G+2T)LexO:GUS line to investigate the impact of the presence of GAGA and TELOBOX motifs in the synthetic promoter. Information about this line is included in New Supplementary Figure 11 and 12.

It is true that the immunoprecipitation efficiency at the LexO region was much higher in the case of BD-VAL1 than that in the case of the BD alone. We have now included results using transgenic plants that we generated before but did not include in the previous version, WT/pLexO::GUS_1/BD-BMI1A and WT/pLexO::GUS_1/BD-RING1B. The immunoprecipitation efficiency in these plants was as high as in the case of BD-VAL1. Since protein amount was not a limiting factor, as all proteins were overexpressed (see new Supplementary Figure 2A), we argue that this could be caused by the fact that VAL1, BMI1A and RING1B can form homodimers (Chen et al., 2020; Satijn & Otte 1999), thus, increasing the immunoprecipitation efficiency. The same result was obtained when analyzing the binding of these proteins in WT/pLexO::GUS_2 background (see New Supplementary Figure 2B). However, despite ChIP results with BD-BMI1A and BD-RING1B showed a similar immunoprecipitation efficiency at LexO region than BD-VAL1, we did not detected incorporation of H2AK121ub or H3K27me3 marks at the reporter locus, neither in WT/pLexO::GUS/BD-BMI1A or BD-RING1B. These negative results support that the effect of BD-VAL1 binding on histone marking is not caused by a higher binding efficiency of BD-VAL1 compared to the other proteins.

Point 2. The authors represent H2AK121ub and H3K27me3 levels referred to FLC and H3ac to ACT7, instead of %Input. The reason of this choice is not clarified in the text. This normalization has to be taken into consideration especially when analysing the levels of H3K27me3 in the atbmi1abc/pLexO::GUS/BD-VAL1 plants (Fig 2E). If FLC (or AG?) will be used for normalization, the authors should show that the levels of H3K27me3 in FLC are not affected in the mutant background, as it happens with most of the H2AK121ub/H3K27me3 marked genes in this mutant.

RESPONSE: ChIP results using anti-H2AK121ub and anti-H3K27me3 antibodies in the WT background were normalized to the levels of these marks at FLC, which is an internal positive control for these modifications and therefore can correct for possible differences in immunoprecipitation efficiency among samples. Similarly, H3ac levels marks at the reporter locus were normalized to the levels of H3ac at *ACTIN 7* (*ACT7*), which is an expressed gene that contains H3ac activation hallmarks and lacks PcG marks (see Supplementary Figure 5). On the other hand, the levels of H3K27me3 in the *atbmi1 abc* background were normalized to the levels of these marks at *AGAMOUS* (*AG*). *AG* is an only H3K27me3 marked gene and the levels of these marks are not altered in *atbmi1 abc*. This is not the case for *FLC*, as it is marked with both H2AK121ub and H3K27me3 and loses both marks in *bmi1 abc* mutants. This information has been included in the new version of the manuscript (see Results section and Supplementary Figure 5). In addition, to support that these modifications are not altered at these control genes in the different transgenic plants, we have included qRT-PCR analyses showing that the expression of these genes is not altered (see Supplementary Figures 5, 8, 13 and 16). In any case, in the new version of the manuscript we have included all ChIP-qPCR data represented as % INPUT in the supplementary information (see Supplementary Figures 3, 4, 9, 14, 15 and 17), showing similar results.

Point 3. The authors show that many TFs, including VAL1, can recruit PRC2 through the EAR domain. To explore if the EAR domain is the point of recruitment of PRC2 and HDAC (through TPL/TPR or SAP18), they transformed plants with the pLexO::GUS/BD-KNU(-EAR) construct. They see a reduction of levels of H3K27me3 (Fig 4B), but also a low protein abundance compared to the other BD-KNU (Suppl Fig 2). Therefore, the reduction of H3K27me3 could be because a lower amount of the BD-KNU(-EAR), not due to the lack of activity in the absence of the EAR domain. Other TFs, or even VAL1 without the EAR domain could be interesting to test to reinforce this point. In fact,

the BD-VAL1(-EAR) construct could be used also to address the point number 1, as it is expected to have the same binding efficiency than BD-VAL1 but should not be able to attract PRC2 activity.

RESPONSE: We again apologize because we did not present appropriate WB results in the previous version of the manuscript. Previous WBs were made just to check presence/absence of the fusion protein in transgenic plants, without taking into consideration the amount or type of tissue used to extract the proteins (e.g. sometimes total proteins were extracted from rosette leaves and other times from seedlings). We did not consider that this was important assuming that if the proteins were overexpressed, some differences in their levels would not represent a limiting factor for their binding. We have now compared WB results from the same and similar amount of tissue (see all WBs in Supplementary Figures). In particular, we have included a new WB showing the expression of BD-KNU and BD-KNU(-EAR) proteins in extracts from 10 seedlings at 10 DAG (see Supplementary Figure 13B).

As the reviewer indicates, BD-VAL1(-EAR) could have be used for the analysis, as it should have the same binding efficiency than BD-VAL1; however, we thought that results using BD-VAL1(-EAR) would be difficult to interpret, as VAL1 interacts with both PRC1 and PRC2, and both VAL1 and PRC1 participate in PRC2 recruitment (see Results line 230-250 and Figure 2F).

Point 4. The authors double verify the EAR domain role of attracting PRC2 by using a BD-EAR construct, but again there is a big difference when protein abundance is compared: BD-EAR amounts are many orders of magnitude higher than BD-KNU. This does not exclude the role of the EAR domain recruiting PRC2, but the higher efficiency of BD-KNU (that is able with lower amounts to induce higher levels of H3K27me3) could mean that other domains of the TF can also participate in the recruitment of PRC2.

RESPONSE: Again, WB result showing detection of DB-EAR protein was not comparable to that of BD-KNU. In this case, in addition to check DB-EAR overexpression, we wanted to know whether it was transported to the nuclei, therefore, we used a nuclear protein extract for WB analysis. We have now included a WB showing BD-EAR amount in seedlings (see Supplementary Figure 14A). Furthermore, we also included LexA BD ChIP results to compare the binding efficiency of BD alone and BD-EAR. The result showed a similar binding efficiency of the two proteins (see Supplementary Figure 15A).

Nevertheless, indeed we observed a higher efficiency in the incorporation of H3K27me3 marks in the case, for instance, of BD-KNU than in that of BD-EAR. As the reviewer indicates, this could mean that other domains of the TF can participate in the recruitment of PRC2. In fact, direct interaction of different PRC2 components with some of these EAR-containing TFs has been reported. We have contemplated and discussed this possibility in the new version (See the discussion section line 435-451).

Point 5. The model proposed by the authors will be strengthened if the role of TPL/TPR and SAP18 connecting VAL1/TF with PRC2 could be tested using their in vivo system. One possibility to explore is to use the BD constructs in TPL/TPR or SAP18 defective mutant background.

RESPONSE: As the reviewer indicates, the role of TPL/TPR or SAP18 connecting VAL1/TF with PRC2 could be explored using our system. However, the initial aim of this work was to understand the role of different TFs in PRC1 and PRC2 recruitment. Our results, in the context of data obtained in other works, has led as to propose this connection. Since we found a considerable amount of data in the literature demonstrating the interaction of TPL/TPR or SAP18 and EAR-TFs, TPL/TPR or SAP18 and HDAs and TPL/TPR or SAP18 and PcG proteins (see references in the manuscript), we thought that the generation of these constructs would not provide novel information. On the other hand, introducing the system in loss-of-function TPL/TPR or SAP18 mutants would be a time consuming project, as it would require generating high-order mutant transgenic plants. I hope that the reviewer will understand that we do not wish to generate these plants.

Reviewer #2:

Point 1. The authors anchor various constitutively overexpressed transcription factors to a synthetic regulatory element in the context of the 35S promoter and assess effects on GUS reporter expression and histone modifications. In this system, it is impossible to detect which events are directly caused by the factor of interest. Once Val1 recruits H2Aub and gene silencing occurs, this may secondarily lead to H3K37me3 and H3 deacetylation. This applies to all Figures. Although they repeat the Val1 recruitment in atbm1abc - they do not show what the H2AK121Ub or H3ac level is in the mutant background, precluding any conclusions about how these events are

linked. The types of conclusions drawn here can only be obtained with conditional gain-of-function TFs that allow a time course analysis to distinguish direct from indirect effects.

RESPONSE: The reviewer indicates that the conclusions drawn in our work can only be obtained with conditional gain-of-function TFs that allow a time course analysis to distinguish direct from indirect effects. However, the work presented in this manuscript has been designed based on a large number of previously published results, both by our group and by other groups. As we mentioned in the general introduction and when introducing the different experiments, direct interaction of most of these factors with one or more that one of the histone modifying complexes that we investigated has been already reported. For example, interaction of VAL1 with PRC1, PRC2 or HDAC, the same for several EAR-containing TFs with TPL/TPR or SAP18, TPL/TPR or SAP18 with HDA or PcG proteins, TFs with PcG proteins... etc. In addition, there are data supporting the direct implication of many of these factors on histone marking. The aim of our work was to understand how PRC1 and PRC2 recruitment occurs and to determine the degree of interdependency among these complexes and possibly with other repressive complexes. To do this, we decided to build a synthetic system that allowed us to assemble the pieces of the puzzle that although present were not yet connected. Therefore, in our opinion, the results of this work within the context of the current knowledge in the field strongly supports that the events we observed are directly linked. In any case, we agree that time course experiments would be interesting to address many other open questions.

As the reviewer suggested, we have now included in the revised version of the manuscript the levels of H2AK121ub marks at the reporter locus in bmi1abc/pLexO::GUS/BD-VAL1 plants (see Supplementary Figure 4A), which we agree it was an important missing piece of information.

Point 2. It seems from the methods, that the authors characterized only one transgenic line per transgene construct and that the data in the figures is derived from that line. We have no idea which if any constructs are functional. Moreover, the amount of the proteins present for the different transfactors varies widely (Fig. S2). KNU -EAR seems to be present at less than 10% of full-length KNU, which may explain the reduced H3K27me3 and increased H3K27ac in those plants relative to full-length KNU - rather than the lack of the EAR domain. On the other hand, some silencing tests rely on endogenous factors (GA and telobox experiment). There is no comparison at all possible for the effect of endogenous factor recruitment compared to the overexpressed transfactors. This experimental setup renders the small differences seen in many of the experiments meaningless unless they are normalized over protein levels or - independent transgenic lines are compared that have a similar expression levels.

"We have no idea which if any constructs are functional".

RESPONSE: We apologize as we did not explain clearly the transgenic lines that were used in this work. This has been now clarified in the new version. In addition, we have included new information and experiments in order to better characterize the different lines and the functionality of the constructs (see results section of the new version and Supplementary information).

We also apologize because we did not present appropriated WB results in the previous version of the manuscript. Previous WBs were made just to check presence/absence of the fusion protein in transgenic plants, without taking into consideration the amount or type of tissue used to extract the proteins (sometimes total proteins were extracted from rosette leaves and other times from seedlings). We did not consider that this was important assuming that if the proteins were overexpressed there may be an excess of protein, and therefore some differences in the levels would not represent a limiting factor for their binding. We have now compared WB results from the same and similar amount of tissue (see all WBs in Supplementary Figures).

We wanted to address whether different recruiting factors could collaborate in H3K27me3 marking. As introducing two different BD fusion proteins in the same plant could result in competition for the binding site, to test this, we inserted into the synthetic promoter a DNA fragment containing the binding motif of TFs previously related with PcG recruitment. BPC1 and AZF1 TFs has been already shown to bind GAGA and TELOBOX motifs, respectively, and to recruit PRC2 (Xiao et al., 2017; Hecker et al., 2015). Although this experiment relies on the binding of endogenous TFs to the synthetic promoter, which may be not expressed in all tissues and whose expression levels most probably do not reach the levels of the overexpressed factor, we wanted to investigate in any case if this had any effect in the global levels of H3K27me3 at the reporter locus and GUS activity. Therefore, we introduced into the reporter construct a fragment of DNA containing one GAGA and two TELOBOX motifs upstream the LexO to generate WT/p(G+2T)LexO::GUS lines. We compared GUS activity levels in WT/p(G+2T)LexO::GUS_1 and

WT/pLexO::GUS_1 homozygous plants, detecting decreased levels in WT/p(G+2T)LexO::GUS_1 seedlings. Nonetheless, it could be argued that the decreased levels of GUS activity in WT/p(G+2T)LexO::GUS might be due to a worse performance of the promoter after the insertion of an extra DNA fragment. However, when comparing the levels of H3K27me3 at GUS reporter locus in the two lines, we detected some levels of H3K27me3 marks in WT/p(G+2T)LexO::GUS_1 but not in WT/pLexO::GUS_1, supporting that TFs recognizing these motifs could mediate PRC2 recruitment. Due to the reasons indicated above, we of course did not expect a perfect correlation between histone marks and GUS activity levels when comparing the results with the two reporter locus (in other words, we did not expect that the binding of 2-3 factors showed 2-3 times higher effect of the binding of 1 factor). However, we found that the incorporation of these cis-regulatory elements into the promoter indeed has an effect on H3K27me3 incorporation and gene expression. This has been also discussed in the new version of the manuscript.

Point 3. To shore up the conclusion that "Dissection of PRC1 and PRC2 recruitment in Arabidopsis connects EAR repressome to PRC2 anchoring" one would expect the authors should have to tested physical interactions between the transfactors and PRC1 and/or HDAC components using full-length and various deletion constructs. Xiao et al. already identified interactions between PRC2 and TOE1 and AZF1 that are mediated by protein domains other than the EAR motif containing domain - in conflict with the model presented here.

RESPONSE: As we mentioned in the manuscript, both in vivo and in vitro interaction of many of these factors has been already reported (for instance, VAL1-BMI1s, VAL1-HDAs, VAL1-SAP18, EAR TFs-TPL/TPR, EAR TFs-SAP18, TFs-PRC2....). Some of these experiments were done using different parts of the proteins (see references in the manuscript). Therefore, we wanted to go one step forward and understand how all these data fitted.

Our results show that BD-EAR by itself was able to recruit PRC2 and HDACs as did the TFs tested. Nevertheless, changes in histone marks levels were much stronger after the binding of full-length TFs than after BD-EAR binding, suggesting that something else may reinforce the recruitment when a full-length TFs is bound. Interaction of EAR TFs with TPL/TPR or SAP18 and TPL/TPR or SAP18 with EMF1 or MSI1 has been demonstrated by different methods including in vivo Co-IP and Mass Spectrometry (see for instance Qüesta et al., 2016, Liu et al., 2014 and Bloomer et al., 2020). Conversely, no single protein motif seems to be responsible for these TFs-PRC2 components interactions (see for instance Xiao et al., 2017, Sun et al., 2019, Xu et al., 2019....), which was puzzling and it was one of the questions we wanted to address. Our data point to an indirect interaction of TFs and PcG proteins via TPL/TPR or SAP18; however, they also suggest that other possible contacts between TFs and different PcG proteins may reinforce PRC2 recruitment, which is not in conflict with previous reports at all. We have discussed this point in the new version of the manuscript (see Discussion section).

Point 4. Why did the authors use a piece from the *ABI3* 5'UTR to test GA repeat and telobox activity? The fragment they used is from the *ABI3* 5'UTR just upstream of the ATG and is cloned in the reverse orientation next to the LEXA binding site. This is very strange - no internal control is provided to assess how introducing this 100 bp piece of DNA alone affects the expression or histone marking of this new synthetic promoter, for example by mutating the GA and telobox motifs it contains. Thus, not only do some assays rely on overexpressed and other on endogenous trans factors, but the artificial promoter for the latter is entirely different form that used for the former. This precludes all comparisons. This paper is not about endogenous recruitment sites and cis motifs but about activity of transfactors. This could have been tested using AZF1 and BPC1 overexpressed in the same manner as all other transfactors tested here. Using lines with equal protein levels of each factor, one can then at least compare H3K27me3 levels caused (directly or indirectly) by factor tethering.

RESPONSE: The source and orientation of the fragment is irrelevant. We chose this fragment from the regulatory region of *ABI3* because it has these two types of motifs relatively close one to the other. The fact that it was cloned in the reverse orientation was by chance. We cloned a blunt fragment that resulted to be in reverse orientation. To date, it has not been reported a requirement of a specific localization/order of these motifs for PRC2 recruitment. In fact, they have been found in different location/distribution at the promoter of PcG targets, therefore, we were not looking for a specific orientation of the motifs, we just wanted them to be present. We have reduced the information about the source and orientation of the fragment to avoid confusion.

In any case, it could be argued that the incorporation of this fragment into the synthetic promoter may result in a worse performance of the promoter. However, when comparing the levels of H3K27me3 at GUS reporter locus in the two lines, we detected some levels of H3K27me3 marks in WT/p(G+2T)LexO::GUS_1 but not in WT/pLexO::GUS_1, supporting that TFs recognizing these motifs can mediate PRC2 recruitment.

As we indicated in the response to point 2, we wanted to address whether different recruiting factors could collaborate in H3K27me3 marking. Therefore, overexpressing AZF1 and BPC1 in the same manner as all other trans-factors will not address this question. On the other hand, introducing two different BD fusion proteins in the same plant could result in competition for the binding site. Therefore, to test this, we inserted these motifs into the synthetic promoter. It is true that this experiment relies on the binding of endogenous TFs to the synthetic promoter, which may be not expressed in all tissues and which expression levels most probably do not reach the levels of the overexpressed factor. However, we wanted to investigate in any case if this had any effect in the global levels of H3K27me3 at the reporter locus and GUS activity, and indeed, we found an effect. We have now explained and discuss these experiments in the new version of the manuscript.

Point 5. Not surprisingly, given the points above, there does not seem to be a linear relationship between H3K27me3 accumulation and reporter assays. In Fig. 2B VAL1 recruitment leads to ca. 0.8 units of H3K27me3 relative to FLC and a 50% reduction in GUS expression (Fig. 2D). In Fig. 3C, a very similar H3K27me3 level after recruitment of KNU, ERF10 over FLC instead leads to an 84% reduction in GUS expression. Finally, in Fig. 4 VAL1 recruitment causes 2 units of H3K27me3/FLC, and again only 50% reduction in GUS. p(G+2T), which aims to recruit endogenous BPC and Zn Finger proteins yields 1 unit of H3K27me3/FLC but only 20% reduction of reporter expression.

RESPONSE: We never expected a linear relationship between H3K27me3 and reporter assays when comparing the performance of different factors. First, it is important to take into consideration that while VAL1 recruit PRC1 and PRC2 for H2AK121ub and H3K27me3 marking respectively, the other factors only recruit PRC2. It has been shown that average expression levels of H2AK12ub/H3K27me3 marked genes are higher that only-H3K27me3 genes, independently on the levels of H3K27me3 (Zhou, Campero-Romero et al., 2017; Kralemann et al., 2020; Yin et al., 2021). Second, this is a synthetic system in which we fuse the LexABD to different factors. This can differentially affect the performance of these factors. Therefore, we compare whether incorporation/removal of the marks was significant in relation to control samples in each case, but we cannot consider quantitative comparisons among factors. The only case that we can compare marks levels and GUS expression between factors is when we use different versions of the same factor (native or mutated KNU or BD and BD-EAR). In addition, as we explained in response to point 2 and 4, in the experimental approach using p(G+2T)lexO::GUS, we cannot expect that the binding of 2-3 factors showed 2-3 times higher effect of the binding of 1 factor. In summary, our system cannot be taken as a quantitative assay for the comparison of different factors.

<u>Reviewer #3:</u>

Point 1. line 141: it should be described what are the criteria to select "appropriate lines", are only single copy insertions identified, analysed and combined? How were the lines selected that are used throughout the manuscript? How many lines were independently analysed? This is also relevant for all the BD lines.

RESPONSE: We apologize as we did not explain clearly the transgenic lines that were used in the previous version of the manuscript. This has been now clarified (see new results section and New Supplementary information). To ensure that the reporter locus was in the same chromatin environment in all cases, each BD overexpressing line was crossed to WT/pLexO::GUS_1 line. Nevertheless, to investigate possible variations in a different chromatin environment, some of these constructs were also crossed to WT/pLexO::GUS_2 line (those results have been included in the new version). We also generated WT/p(G+2T)LexO:GUS line to investigate the impact of the presence of GAGA and TELOBOX motifs in the synthetic promoter. Information about this line is included in the new version.

Point 2. figures 1C/D: the %IP for BD-VAL1 is much higher than just BD, which may reflect differences in the lines (number of transgenes)? As these strong differences exist, this may also have an influence on H2AUbi and H3K27me3 recruitment. The analyses should be provided for at least two independent transgenic lines.

RESPONSE: In all cases, a line containing a single transgene were selected. This has been now indicated in the new version of the manuscript. However, it is true that the immunoprecipitation efficiency at LexO region was much higher in the case of BD-VAL1 than that in the case of BD alone. We have now included results using other transgenic plants, as is the case of WT/pLexO::GUS_1/BD-BMI1A and BD-RING1B. The immunoprecipitation efficiency in these plants was as high as in the case of BD-VAL1. Since protein amount was not a limiting factor, as all proteins were overexpressed (see new Supplementary Figure 2A), we argue that this could be caused by the fact

that VAL1, BMI1A and RING1B can form homodimers (Chen et al., 2020; Satijn & Otte 1999), thus, increasing the immunoprecipitation efficiency. The results with these plants support that the effect of BD-VAL1 binding on histone marking is not caused by a higher binding efficiency of BD-VAL1 compared to the other proteins.

Point 3. In addition, the different transcription factor lines show very different protein level (sup fig 2), and likely are therefore hardly comparable. For instance, BD-KNU (-EAR) is expressed at much lower levels than BD-KNU (sup fig 2). The authors show that BD KNU EAR represses much weaker than BD KNU (fig 4D) - how can the authors exclude that this is just a function of different protein level in the different constructs?

RESPONSE: We apologize because we did not present appropriate WB results in the previous version of the manuscript. Previous WBs were made just to check presence/absence of the fusion protein in transgenic plants, without taking into consideration the amount or type of tissue used to extract the proteins (sometimes total proteins were extracted from rosette leaves and other times from seedlings). We did not consider that this was important assuming that if the proteins were overexpressed there may be an excess of protein, and therefore some differences in the levels would not represent a limiting factor for their binding. We have now compared WB results from the same and similar amount of tissue (see all WBs in Supplementary Figures). In particular, we have included a new WB showing the expression of BD-KNU and BD-KNU(-EAR) proteins in extracts from seedlings at 10 DAG (see Supplementary Figure 13B).

Point 4. ChIP data are always normalized to *FLC* or *ACT7* - is the expression of these genes affected by the transgenes? Raw data (% of IP) should be shown for the ChIP experiments, so that it is possible to comment on the quality and comparability of the ChIP experiments.

RESPONSE: ChIP results using anti-H2AK121ub and anti-H3K27me3 antibodies in WT background were normalized to the levels of these marks at *FLC*, which is an internal positive control for these modifications and therefore can correct for possible differences in immunoprecipitation efficiency among samples. Similarly, H3ac levels marks at the reporter locus were normalized to the levels of H3ac at *ACTIN 7* (*ACT7*), which is an expressed gene that contains H3ac activation hallmarks and lacks PcG marks. On the other hand, the levels of H3K27me3 in atbmi1abc background were normalized to the levels of these marks at *AGAMOUS* (*AG*). *AG* is only marked with H3K27me3 and the levels of these marks are not altered in *atbmi1 abc*. This is not the case of *FLC*, as it is marked with both H2AK121ub and H3K27me3 and loses both marks in *bmi1 abc* mutants. This information has been included in the new version of the manuscript (see results section and Supplementary Figure 5). To support that these modifications are not altered at control genes in the different transgenic plants, we have included qRT-PCR analyses showing that the expression of these genes is not altered (see Supplementary Figures 5, 8, 13, 16). In any case, in the new version of the manuscript we have also included all ChIP-qPCR data represented as % INPUT in the supplementary information (see Supplementary Figures 4, 8, 13, 14 and 16), showing similar results.

Point 5. LexA BD recruitment is to regions 1-3 (where the LexA binding site is) (fig 1) but histone marks are recruited to region 4 - (e.g. fig 2) - how is this explained? Is this a specific feature of the used GUS gene? Thus, further regions in the transgene (5´ of the promoter should be analysed and ideally transgenes without the GUS gene should be analysed.

RESPONSE: The binding of the fusion proteins displays its maximum at LexO region (position 2), which makes sense as it is where the DNA sequence recognized by LexA BD is located. The fact that we observed some enrichment at adjacent regions is because in ChIP experiments chromatin is randomly disrupted in 500-200 bp fragments by sonication, thus, you can have different fragments containing the LexO together with one or another flanking region that can be also amplified. This is also the case for histone marks ChIP experiments. In these experiments, we checked the 5´promoter region (position 1), LexO region (position 2), 5´region of GUS reporter (position 3), middle part of GUS reporter (position 4) and 3´region of GUS reporter. Although we did not scan the complete region of the reporter locus as this work was not directed to analyze the distribution of the marks (for genome-wide distribution analysis of H2AK121ub and H3K27me3 at genes see Zhou, Romero-Campero et al. 2017), the results are in concordance with the genome wide distribution of these marks, as PcG complexes are recruiter to target promoters and incorporate their marks at gene body regions.

Point 6. GUS activity is shown as an average of different seedlings. It seems that only one replicate was performed - how many independent experiments were performed? Also stained seedlings should be shown to reveal that GUS expression is present in the same tissues.

RESPONSE: GUS activity is shown as an average of different seedlings and experiments. This is now specified in the figure legends (see new version of the manuscript). We have in addition included GUS staining pictures of pLexO::GUS lines and gene expression analysis of GUS reporter (see new Supplementary Figure 1). GUS expression was also checked in several transgenic lines to show that GUS activity correlated with gene expression levels (see new Supplementary Figure 6).

TPC2020-RA-00689R1 2nd Editorial decision – *revision requested* March 19, 2021

As you will read below, only one of the previous reviewers is fully content with the revisions that you made. Previous Reviewer 3 wants an explanation for the distance of the histone modification peak from the binding site of the modifier. We felt that this should at least be discussed. Reviewer 2 has a whole range of concerns that has not been erased by the changes and requests a thorough revision of the text in several paragraphs, so as not to over-interpret the findings. He/she especially does not accept the quantitative comparisons based on the ChIP data that were not normalized to input or to the histone occupancy as such. The statement of synergism in PRC2 recruitment is not well supported and should be removed, it appears rather additive. We felt that these issues need to be addressed. In addition, please give credit to earlier work by referring to the mentioned publication and answer the remaining concerns.

-- Reviewer comments:

Reviewer #1 (Comments for the Author):
The authors have appropriately addressed my questions. An important amount of clarifications and control data have been added to the new version of the manuscript.
This version has no amendments to be included from my side.

Reviewer #2 (Comments for the Author):
The authors have made some changes to the manuscript, which have improved it. However, they have not adequately addressed my concerns. To be quite honest, the manuscript text could be described as misleading or even manipulative in parts. The text should represent the findings, no more and no less.

The findings are as follows- two artificial recruitment systems were made. One containing Lex O, the other containing LexO and a region from the 5'UTR of the *ABI3* gene, which contain GA repeats and a telobox.

To these fragments diverse factors were recruited that were overexpressed (VAL1, FLC, ERF10, KNU, KNU without EAR, EAR alone, BD alone, EMF1) or endogenous factors were recruited (BPC1, AZF1/TRBs).

What the data shows is that all recruited factors except BD alone did lead to significant increased H3K27me3 and reduced GUS expression, but the vast majority (except VAL1) had no effect on H2Aub. The combined data suggests a contribution of the EAR domain and of other protein domains in PRC2 recruitment. The EAR-dependent mechanisms could be via TPL/HDAC or independent of these factors. Since a role of TPL or HDACs in PRC2 recruitment was not tested in their tethering system whether the recruitment is via these factors remains to be ascertained.

1. In light of the data as they stand the title, the abstract and the second summary statements need revision.
Title: Dissection of 1 PRC1 and PRC2 recruitment in Arabidopsis connects EAR repressome to PRC2 anchoring
One sentence summary: PRC2 can be recruited to target genes independently of PRC1 by EAR-TFs. The EAR domain, through its interaction with TPL/TPR1-4 corepressors or SAP18, acts as a docking point for PRC2 and HDACs.

Abstract: Interestingly, all these TFs contain an EAR domain that acts as docking point for PRC2 and HDACs, connecting two different repressive mechanisms. Furthermore, we show that different TFs act synergistically in PRC2 anchoring, which may be required to maintain a long-term repression.

I am happy to detail my specific objections to these statements and how they either are not supported by the data provided as stated or had already been shown by others, but likely this will become clear in my additional major

points below.
2. In addition, I strongly recommend (together with all other reviewers) that the authors show the input normalized (over H3 or H2A would even be better to account for possible histone loss) data for the histone modifications in the main text. For the KNU without EAR the H3K27me3 looks significantly increased over BD in the supplement (fS14A); this ChIP data seems to have a higher variance after normalization over FLC.
3. The authors should either not at all quantitatively compare changes in H3K27me3 and GUS expression derived from overexpressed or endogenous factors and from binding to different templates, but limit themselves to significant changes or not (preferred). This will avoid a lot of the subjective data interpretation in the text (on example is comparison BD+EAR alone and the endogenous factors binding G+T - very similar data, very different discussion of the data in the results, and title/abstract/summary sentence). If the authors wish to make quantitative comparisons (which I would strongly advise against) they need to point out all the caveats why these are not directly comparable at the same time.
4. Since it is highly unlikely that the response of the endogenous factors to the GT recruitment template is saturated, recruiting another overexpressed factor to the same DNA that furthermore links to PRC1 and to PRC2 is not at all an indication of cooperativity. The discussion of the data and the abstract should reflect this.
5. The authors need to state when they introduce Fig. 4A- that the presence of EAR domains in 3 PRC2 recruiting factors (BPC1, AZF1, ZAT6 and TOE1) had already been pointed out by others (Xiao et al. 2017) and that domains outside these EAR motifs were shown to be sufficient for direct physical interaction between these and PRC2 core complex components by that paper.
6. It is not clear to me why the authors look for enriched PRC2 recruitment motifs in promoters and not under PRC2 bound sites. The latter are known to flank the TSS and extend into the transcribed region.

The revised manuscript has several typos, including in section headings (line 256) and should be revised for spelling and grammar.

Reviewer #3 (Comments for the Author):
Overall, the authors answered most of my questions and concerns.
Only comment 5." LexA BD recruitment is to regions 1-3 (where the LexA binding site is) (fig 1) but histone marks are recruited to region 4 - (e.g. fig 2) - how is this explained?" has not been properly addressed.

While the sonication efficiency explains some broader distribution, it does not explain why the peak is at a different site (at LexA (for construct) or GUS gene (for histone mark)). This must rely on a biological not technical phenomenon.

TPC2020-RA-00689R2 2nd Revision received April 6, 2021

Reviewer comments and **author responses**:

Reviewer #1:

Point 1. The authors have appropriately addressed my questions. An important amount of clarifications and control data have been added to the new version of the manuscript. This version has no amendments to be included from my side.

RESPONSE: Thank you.

Reviewer #2:

Point 1. In light of the data as they stand the title, the abstract and the second summary statements need revision.

Title: Dissection of PRC1 and PRC2 recruitment in Arabidopsis connects EAR repressome to PRC2 anchoring

One sentence summary: PRC2 can be recruited to target genes independently of PRC1 by EAR-TFs. The EAR domain, through its interaction with TPL/TPR1-4 corepressors or SAP18, acts as a docking point for PRC2 and HDACs.

Abstract: Interestingly, all these TFs contain an EAR domain that acts as docking point for PRC2 and HDACs, connecting two different repressive mechanisms. Furthermore, we show that different TFs act synergistically in PRC2 anchoring, which may be required to maintain a long-term repression.

RESPONSE: According to Reviewer comments, we have revised the running title, the one sentence summary, the abstract and the manuscript text in order to do not over-interpret our findings. However, we did not modify the title since our data indeed show a connection between the EAR repressome (EAR-containing TFs) and PRC2 anchoring that has not been proposed before, despite the fact that in addition to the EAR domain other domains within these TFs can contribute to PRC2 recruitment (as has been discussed in the manuscript). We hope that Reviewer will agree.

Point 2. In addition, I strongly recommend (together with all other reviewers) that the authors show the input normalized (over H3 or H2A would even be better to account for possible histone loss) data for the histone modifications in the main text. For the KNU without EAR the H3K27me3 looks significantly increased over BD in the supplement (fS14A); this ChIP data seems to have a higher variance after normalization over FLC.

RESPONSE: According to Reviewer indications, we have placed within the main Figures ChIP-qPCR results normalized to input and moved to Supplementary Information the results normalized to an internal control (see new version).

Point 3. The authors should either not at all quantitatively compare changes in H3K27me3 and GUS expression derived from overexpressed or endogenous factors and from binding to different templates, but limit themselves to significant changes or not (preferred). This will avoid a lot of the subjective data interpretation in the text (on example is comparison BD+EAR alone and the endogenous factors binding G+T - very similar data, very different discussion of the data in the results, and title/abstract/summary sentence). If the authors wish to make quantitative comparisons (which I would strongly advise against), they need to point out all the caveats why these are not directly comparable at the same time.

RESPONSE: We have followed the Reviewer's indications (see page 9-10 of the new version and new Figure 3).

Point 4. Since it is highly unlikely that the response of the endogenous factors to the GT recruitment template is saturated, recruiting another overexpressed factor to the same DNA that furthermore links to PRC1 and to PRC2 is not at all an indication of cooperativity. The discussion of the data and the abstract should reflect this.

RESPONSE: We apologize for not clearly explaining this point. We did not want to indicate cooperativity but rather an additive effect. We hope it has now become clear throughout the manuscript in the new version.

Point 5. The authors need to state when they introduce Fig. 4A- that the presence of EAR domains in 3 PRC2 recruiting factors (BPC1, AZF1, ZAT6 and TOE1) had already been pointed out by others (Xiao et al. 2017) and that domains outside these EAR motifs were shown to be sufficient for direct physical interaction between these and PRC2 core complex components by that paper.

RESPONSE: We have included this and other related references when introducing Figure 4A (line 340-341). In addition, the fact that other regions of BPC1, AZF1, ZAT6 and TOE1 outside the EAR have been shown to interact with different PRC2 components have been also discussed (see line 454-461 in page 14).

Point 6. It is not clear to me why the authors look for enriched PRC2 recruitment motifs in promoters and not under PRC2 bound sites. The latter are known to flank the TSS and extend into the transcribed region.

RESPONSE: We apologize for not clearly describe the analyzed region. We analyzed the 500 bp region upstream the start codon (ATG). We named this region "proximal promoter" (page 4 of the previous version, first paragraph of the results section). However, we agree with the Reviewer that this term can lead to misinterpretation; therefore, we now indicate "the 500 bp region upstream the ATG". We restricted the search to this region since, as the Reviewer indicates, it has been shown to be bound by Polycomb Group components in different reports.

Point 7. The revised manuscript has several typos, including in section headings (line 256) and should be revised for spelling and grammar.

RESPONSE: We apologize for this. We have now tried to correct typos and revised the manuscript for spelling and

grammar.

Reviewer #3:

Point 1. Overall, the authors answered most of my questions and concerns. Only comment 5." LexA BD recruitment is to regions 1-3 (where the LexA binding site is) (fig 1)but histone marks are recruited to region 4 - (e.g. fig 2) - how is this explained?" has not been properly addressed. While the sonication efficiency explains some broader distribution, it does not explain why the peak is at a different site (at LexA (for construct) or GUS gene (for histone mark)). This must rely on a biological not technical phenomenon.

RESPONSE: We apologize for not clearly describing the exact position of the primer pairs used for qPCR analysis, which lead to a wrong interpretation of the results. We have now included the exact localization of the primer pairs within the synthetic locus (see new Figure 1, 2 and 3), and explained why we used these primer pairs (see paragraph 181-184 and 196-201 in page 6). Although we used the 5 primer pairs in all the analysis, primer pairs at position 1, 2 and 3 were designed to analyze the binding of the fusion proteins to the synthetic promoter, and primer pair at position 4 was designed to check the presence of histone marks at the gene body. Based on genome wide distribution studies (Zhou et al., 2017; Yin et al., 2021), if these marks are present, they should be detected at gene body (position 4), although not necessarily displaying at this location their highest levels. The purpose of these experiments was not to determine the distribution of the histone marks within the reporter gene, but rather to check presence/absence of these marks.

In any case, the enrichment of BD-proteins at LexO and the detection of the histone modifications at the reporter gene body is consistent with the prevalent idea that Polycomb Group complexes are recruited to a specific regulatory region from which they incorporate their marks at target gene bodies. We have discussed this in the new version (paragraph 213-217 in page 7).

TPC2020-RA-00689R2 3rd Editorial decision – *accept with minor revision* May 4, 2021

Thank you for your email about the status of the paper. As it turns out, one of the editors had missed a notification, so the decision had indeed been stalled, so your email was important to bring our attention to the issue. Please accept my sincere apologies for that delay.

We have received a re-review of your manuscript entitled "Dissection of PRC1 and PRC2 recruitment in Arabidopsis connects the EAR repressome to PRC2 anchoring." On the basis of the advice received, the board of reviewing editors would like to accept your manuscript for publication in The Plant Cell. This acceptance is contingent on revision based on the comments of the reviewer. In particular, please consider the following. The remaining concern of the reviewer is the claim for direct "recruitment", and he/she is right that you do not show data to support that. But by changing the title, the short title, and the one-sentence summary, this problem can be solved. Here are our suggestions

Title: EAR domain-containing transcription factors establish PRC2-mediated chromatin modifications
Short title: EAR domain transcription factors engage PRC2 activity
One-sentence summary: PRC2 activity is directed to target genes by EAR domain-containing transcription factors, independently of PRC1.

Of course, it would be necessary to delete the claims for "recruitment" also throughout the manuscript. Please do this carefully and consider or modify the suggestions. Please provide an additional file with all changes indicated, so that a rapid evaluation of the revision is possible.

Reviewer #2 (Comments for the Author):

The revised manuscript is significantly improved and reads very well. I am still a bit concerned about the title, short title and two sentence summary. This is because the authors have not studied either PRC2 recruitment or HDAC recruitment; they infer that the chromatin regulators bound the locus assayed at some point by measuring changes in histone modifications after constitutive tethering of transcriptional repressors to a given site. In the absence of direct/causal physical interaction data, this leaves open the possibility of intermediates between the factor tethered

and histone modifiers.

TPC2020-RA-00689R3 3rd Revision received May 6, 2021

Reviewer comments and **author responses**:

Reviewer #2:

Point 1. I am still a bit concerned about the title, short title and two sentence summary. This is because the authors have not studied either PRC2 recruitment or HDAC recruitment; they infer that the chromatin regulators bound the locus assayed at some point by measuring changes in histone modifications after constitutive tethering of transcriptional repressors to a given site. In the absence of direct/causal physical interaction data, this leaves open the possibility of intermediates between the factor tethered and histone modifiers.

> **RESPONSE: According to Reviewer 2's suggestions, we have changed the title, short title and two-sentence summary, and we have clarified throughout the manuscript that the EAR domain TFs when recruited to the synthetic promoter trigger PRC2 and HDA activities. Although we did not show direct evidence, we hypothesized that TPL/TPR1-4 corepressors or SAP18 may act as intermediate factors (proposed model in Figure 5 and discussion), as they have been shown to directly interact with EAR domain-factors, PRC2 components and HDAs.**

TPC2020-RA-00689R3 4th Editorial decision – *acceptance pending* May 6, 2021

We are pleased to inform you that your paper entitled "EAR domain-containing transcription factors trigger PRC2-mediated chromatin marking in Arabidopsis" has been accepted for publication in The Plant Cell, pending a final minor editorial review by journal staff. At this stage, your manuscript will be evaluated by a Science Editor with respect to its presentation of scientific content, compliance with journal policies, and presentation for a broad readership.

Final acceptance from Science Editor May 14, 2021
